# Adolescent Social Capital as a Source of Resilience against Emotional and Behavioural Difficulties in Times of Crisis: Longitudinal Evidence from the COVID-19 Pandemic

**DOI:** 10.1007/s10964-025-02295-5

**Published:** 2025-12-04

**Authors:** James Laurence, Bill Calvey

**Affiliations:** UCL Social Research Institute, University College London, London, UK

**Keywords:** Internalizing and externalizing difficulties, Mental health, Social capital, Stress-buffering, Crises, Resilience

## Abstract

The COVID-19 pandemic precipitated sharp increases in adolescent emotional and behavioral difficulties, highlighting the need for effective psychosocial buffers during large-scale crises. While several protective factors have been identified, the stress-buffering role of social capital remains largely underexplored. This study investigates whether different dimensions of peri-pandemic social capital acted as stress-buffers for adolescent emotional and behavioral difficulties during the pandemic. Using four waves of nationally representative UK panel data (*n* = 1,203 individuals; *M*_*age*_=12.2 years; SD_*age*_=1.74; female = 53%; non-White = 21.5%), this study examined changes in adolescents’ self-reported ‘Internalizing and Externalizing Strengths and Difficulties Questionnaire’ scores from pre-pandemic (2017–18) over three time-points during the first year of the pandemic (2020–21). Social capital was measured across three domains: strong-tie network structure (e.g., family connectivity, close friendships), extracurricular involvement, and neighborhood social capital (adolescent perceived neighborhood safety, the social embeddedness of adolescents’ parents in local networks, and community- (contextual-) level social capital). Multilevel modelling and longitudinal fixed-effects modelling reveal that adolescents with stronger peer and family networks experienced significantly smaller increases in internalizing symptoms. Meanwhile, only perceived neighborhood safety consistently buffered externalizing difficulties. Indicators of parent- and contextual-level neighborhood social capital showed minimal protective effects. Perceived social support explained much, although not all, of why peer/family networks moderated trajectories in internalizing difficulties. However, it explained little of why neighborhood safety buffered externalizing difficulties. These findings suggest social capital can confer protection on adolescent mental health during large-scale crises but also underscore how different dimensions of social capital may be more/less effective in safeguarding particular clusters of emotional-behavioral problems. Crisis-preparedness and youth policy should reflect this outcome conditionality to foster protection via social capital from future crises.

**Supplementary Information:**

The online version contains supplementary material available at 10.1007/s10964-025-02295-5.

## Introduction

The COVID-19 pandemic adversely impacted multiple dimensions of adolescent mental health, such as internalizing and externalizing difficulties, depression, anxiety, and positive mental wellbeing (Magson et al., 2021). While studies suggest that adult mental health has largely recovered post-pandemic (e.g., Reutter et al., 2024), evidence suggests that poorer post-pandemic mental health has persisted among adolescents (e.g., Montero-Marin et al., 2023). Given prolonged experiences of emotional-behavioral difficulties in adolescence can harm mental health long into adulthood (Clayborne et al., 2019), it is important to identify what factors can protect adolescent mental health during large-scale societal disruptions. One protective factor may be social capital – that is, social networks and norms of reciprocity/trust – which can buffer the impact of stressors on mental health (Kawachi & Berkman, 2001). Social capital has emerged as key stress-buffering factor among adults during the pandemic (Laurence et al., 2024; Sato et al., 2022). However, to date, little work has explored its potential stress-buffering role for adolescent emotional-behavioral difficulties. This study examines the protective role that different dimensions of adolescent social capital (friend/family networks, extracurricular involvement, neighborhood social capital) played in cushioning the impact of the pandemic on adolescent-reported emotional-behavioral problems. In addition, the study explores whether any stress-buffering role of social capital can be explained by adolescents’ perceived social support. In doing so, the study sheds light on the role of social resources for protecting adolescents during major crises, and the importance of social capital for youth crises-preparedness policies.

### Social Capital, Stress-buffering and Mental Health

Social capital constitutes social networks and their attendant norms and trust that facilitate cooperation for mutual benefit (Putnam, 2000). It is typically divided into bonding social capital (closer, stronger ties involving a greater frequency of interaction, especially among family and friends) and bridging social capital (constituting weaker ties across social groups, often captured through civic/associational participation) (Morrow, 1999). Social capital can also be contextualized by the setting in which ties are primarily embedded, such as the household, neighborhood or school (Harpham et al., 2002) and can be measured at both the individual level (e.g., personal network size) and the contextual level (e.g., community-level network density, or societal levels of civic engagement) (Laurence & Calvey, in press).

Social capital has long been linked to different indicators of better mental health (De Silva et al., 2005), through mechanisms such as support networks, positive psychological states like belongingness or security, or commitments (and a capacity) to work together to achieve shared goals i.e., collective efficacy (Kawachi & Berkman, 2001). These benefits can become especially important for mental health in times of adversity, when social capital can act as a buffer, protecting individuals from the psychological impact of acute or chronic stressors (Zeng & Wu, 2022). Studies often explain this protective capacity of social capital using the stress-buffering model of social support, which proposes that supportive relationships (both actual and perceived) reduce the negative impact of stressors on health by providing emotional, informational, or practical resources that reduce the perceived threat of, or help cope with, stressors in everyday life (Cohen & Wills, 1985). Based on this model, the social networks that underpin social capital, coupled with their constituent norms of trust/reciprocity, are posited to foster both greater access to social support and perceptions that support would be available if needed, driving social capital’s stress-buffering capacity (Kim et al., 2006).

Recently, research has examined whether this stress-buffering capacity of social capital extends to large-scale crises (Aldrich & Meyer, 2014), such as natural disasters (Lê et al., 2013) or economic recessions (Lindström & Giordano, 2016). In times of significant societal disruption, networks of trust/reciprocity are believed to cushion harm from stressors by enabling better cooperation and providing “information, aid, financial resources and…emotional and psychological support” (Aldrich & Meyer, 2014, p.256). Indeed, research has shown the efficacy of social capital for cushioning the impact of the COVID-19 pandemic on adult mental health. Individuals who reported higher neighborhood connectivity (such as trusting neighbors, or feeling safe walking alone at night) (O’Donnell et al., 2022), who lived in regions (Sato et al., 2022) or countries (Laurence & Calvey, in press) with higher mean stocks of (contextual) social capital, or who had larger friendship networks (Perry et al., 2023) exhibited smaller pandemic increases in distress. These effects were shown to operate via greater perceived support, reduced loneliness, and enhanced psychological resilience (Laurence, 2025b).

### Social Capital Stress-buffering among Adolescents and The COVID-19 Pandemic

While the benefits of social capital for adult mental health are well-established, a growing body of research demonstrates how adolescent social capital may similarly improve adolescent mental health, especially internalizing/externalizing problems (Hirota et al., 2022; McPherson et al., 2014). This includes strong-tie social capital among family (e.g., eating meals together more frequently) (Kameyama et al., 2021) and friends (e.g., number of friends) (Klanšček et al., 2014), which is linked with fewer emotional-behavioral difficulties (although friendships can increase externalizing symptoms, e.g., due to peer deviance). The social capital in adolescents’ neighborhoods (neighborhood social capital) is also linked with positive mental health outcomes. For example, positive perceptions of people in their neighborhood (e.g., perceived neighborhood safety) is linked with fewer adolescent externalizing difficulties (Midouhas et al., 2021), while the social embeddedness of adolescents’ parents in the neighborhood (e.g., parents’ neighborhood belonging) (Aminzadeh et al., 2013), and average levels of social capital (e.g., reciprocity) among all neighbors in an area (contextual social capital) (Turkmani et al., 2023), are both linked with fewer emotional problems. These benefits are evident even after accounting for confounding characteristics linked with both social capital and youth mental health, including adolescents’ gender, age, ethnicity and health (Berkelbach van der Sprenkel et al., 2022; Loeber & Burke, 2011), household socio-economic status, such as parental education, lone-parent status, employment status, and financial stability (Rothon et al., 2012), and levels of socio-economic disadvantage in the wider-community (Aminzadeh et al., 2013).

Despite evidence linking social capital to fewer internalizing/externalizing problems, far less is known about whether adolescents’ social capital (like adults’) can buffer the impact of large-scale crises (e.g., the COVID-19 pandemic) on their emotional-behavioral difficulties. Moreover, it remains unclear whether particular forms of adolescent social capital may be differentially protective for specific domains of adolescent internalizing/externalizing symptomatology. Internalizing and externalizing symptom clusters follow distinct etiologies, contextual sensitivities, and developmental trajectories, which could shape the efficacy of (different types of) social capital for cushioning the impact of large-scale crises. Internalizing difficulties are linked to vulnerabilities such as temperamental emotional reactivity and family emotional climate (such as overcontrolling parenting), and are particularly sensitive to interpersonal contexts, such as limited family support, high conflict, or peer rejection (Achenbach et al., 2016). Externalizing difficulties are linked with deficits in self-regulation and adverse family environments (e.g., harsh or inconsistent parenting), and are also more sensitive to contextual influences outside the home, such as exposure to neighborhood disadvantage or deviant peer affiliations exacerbating behavioral difficulties (Loeber & Burke, 2011). These clusters also exhibit different developmental trajectories, with internalizing symptoms typically increasing across adolescence, especially among girls, and externalizing problems often peaking in childhood/early adolescence, before declining for most (Loeber & Burke, 2011). Research indeed shows different dimensions of adolescent social capital appear differentially associated with internalizing and externalizing outcomes. For example, while neighborhood social capital appears more closely related to externalizing problems, family/peer social capital is related more to internalizing difficulties (Smyth & Darmody, 2021).

Research has begun exploring whether social network characteristics more generally protected adolescent internalizing and externalizing difficulties during the pandemic. The quality of adolescents’ social ties, such as stronger family (although not friend) attachment, was linked with better outcomes, especially internalizing symptoms (Afriat et al., 2023). Adolescents feeling more socially disconnected (e.g., “I feel disconnected from the world around me”) also saw worse pre- to peri-pandemic internalizing outcomes (Magson et al., 2021). One study directly analyzing strong tie network structure found adolescents who spent more time speaking with friends/family experienced less anxiety and depression with the onset of the pandemic (Riazi et al., 2023). Most pandemic research, however, has examined the protective role of perceived social support – one of the key pathways through which social capital is posited to cushion stressors (Wolf & Schmitz, 2024). For example, adolescents who reported higher perceived social support experienced less negative pre- to peri-pandemic trends in emotional-behavioral difficulties (Shoshani, 2024).

These studies provide indicative evidence that social capital could buffer adolescent internalizing/externalizing difficulties during major crises like the pandemic. However, there are key knowledge gaps. Firstly, existing pandemic research largely investigates the protective role of perceived support rather than directly measuring the features of network structure (e.g., size, frequency of interaction) upon which social capital is built. Secondly, studies generally focus on the role of strong-tie (family/close friend) relationships, with other dimensions of adolescents’ social capital (e.g., extracurricular engagement) remaining underexplored. Thirdly, one dimension of social capital that is particularly absent is the role of neighborhood social capital, a key stress-buffering domain among adults during the pandemic. Fourthly, there is no systematic treatment of whether different types of social capital may confer differential protection for adolescents’ internalizing or externalizing difficulties. Taken together, insights into if, which, and how dimensions of adolescent social capital may confer protection for different clusters of emotional-behavioral difficulties during crises remain limited.

## Current Study

Few studies have assessed whether social capital can cushion the impact of large-scale crises on adolescents’ emotional and behavioral difficulties, nor examined the pathways through which any stress-buffering might occur. Using the COVID-19 pandemic as a case study, this study tests the stress-buffering role of social capital for adolescents’ emotional-behavioral problems during large-scale crises. It hypothesizes that adolescents (aged 10–15 years old) with higher peri-pandemic social capital (strong-tie network structure, civic engagement, and neighborhood social capital) will exhibit smaller increases in internalizing and externalizing difficulties across the pandemic period (Hypotheses 1). However, given outlined domain specificity in the etiology, context, and trajectory of adolescent internalizing and externalizing symptoms, it is also hypothesized that different forms of social capital will exhibit different protective capacities for emotional-behavioral difficulties (Hypothesis 2). Strong-tie social capital in families and close friendships may provide comparatively greater protection against internalizing symptoms via better emotional co-regulation, reduced threat appraisals, and shared meaning-making that emerge from supportive family/friend relations. By contrast, given externalizing problems are more sensitive to opportunities, supervision, and local norms, neighborhood social capital may confer more protection by enabling greater informal social control, reinforcing prosocial norms, and supporting more structured socializing. Adolescent civic engagement may afford more widespread protection given structured activities, adult oversight, and prosocial norms may reduce the emergence of behavioral problems while the role of civic engagement in fostering more supportive friendship networks may protect adolescents from internalizing difficulties. Lastly, it is hypothesized that any observed stress-buffering effect of social capital will come through higher levels of perceived social support (Hypothesis 3). To better identify any protective role of social capital the analysis adjusts for adolescent, household and spatial socio-demographic characteristics known to be correlated with both social capital and adolescent emotional-behavioral difficulties.

## Methods

### Data

This study mobilizes data from the UK Household Longitudinal Study, merging data from the UK Household Longitudinal Study *Mainstage* survey and the UK Household Longitudinal Study *COVID-19 Study* (which tracked a sub-sample of households from the *Mainstage* survey during the COVID-19 pandemic). The UK Household Longitudinal Study Mainstage data is a nationally representative household panel survey, which recruited 40,000 households to take part in 2009. The Mainstage data comprises 14 waves, tracking the same households from wave 1 (2009–2010) to wave 14 (2022–2023). The UK Household Longitudinal Study contains an adult sample (all adults in a household aged 16+) and a separate youth sample (comprising all 10–15-year-olds present in the household). Children in a UKHLS household enter the youth sample when they turn 10-years-old and leave the youth sample when they turn 16-years-old, moving into the adult sample. This study focuses on the adolescent/youth sample. The analysis uses wave 9 of the UK Household Longitudinal Study Mainstage data to capture adolescents’ pre-pandemic emotional-behavioral difficulties (January 2017-December 2018), which is the most recent available pre-pandemic wave containing adolescent emotional-behavioral difficulties. Wave 9 of the UK Household Longitudinal Study Mainstage also contains a limited set of adolescent social capital indicators.

The UK Household Longitudinal Study *COVID-19 Study* data follows a sub-sample of participants from the UK Household Longitudinal Study Mainstage survey over the COVID-19 pandemic (April 2020 to September 2021). Paper self-completion surveys were sent to the responsible parent of all 10-15-year-olds present in the household to be completed by their children. These adolescents were surveyed at 3 time-points during the pandemic: August 2020 (29th July-1st September), November 2020 (16th November-18th December), and March 2021 (1st March-9th April). All eligible adolescents (10–15 years-old) were invited to participate in every wave. The response rates for the 3-waves were *n* = 1,411 (44 per cent), *n* = 1,432 (43 per cent), and *n* = 1,388 (50 per cent) respectively, with 66 per cent of adolescents appearing in two or more waves. Testing shows that across wave-pairs, adolescents in more disadvantaged environments (disadvantaged areas; fewer employed in household; household not coping well financially) were more likely not to respond (importantly, measures of emotional-behavioral difficulties did not predict response). All three COVID-19 study waves contain measures of adolescents’ emotional-behavioral difficulties, but full peri-pandemic measures of social capital were only measured in the November 2020 wave.

Together, these data allow us to track pre- to peri-pandemic changes in emotional-behavioral difficulties. The waves of the UK Household Longitudinal Study data are designed to be analyzed as either pooled cross-sections over time or as a longitudinal panel dataset (Kaminska & Lynn, 2024). This study analyses the data as both. Several sample restrictions are in place. Analyses are restricted to adolescents who participated in the November 2020 wave of the COVID-19 study (*n* = 1,432 individuals) who have available peri-pandemic social capital data (potential limitations of this unavoidable restriction are discussed below). *N* = 76 individuals are excluded due to missingness on their key socio-demographic characteristics, and a further *n* = 153 individuals are excluded due to absent address data, resulting in a valid sample of *n* = 1,203 individuals.

When the data are treated as pooled cross-sections, data from all four waves (1 pre-pandemic/3 peri-pandemic) are examined, resulting in an analytical sample of *n* = 3,176 person-observations. Cross-sectional probability weights correct the sample for non-response (see Supplementary-Appendix: S.1 for descriptives of the pooled cross-sectional sample). When the data are treated as longitudinal panel data, only the 2017-18 pre-pandemic wave and November 2020 peri-pandemic wave are used (as repeat social capital measures were only taken in these waves). *N* = 627 individuals participated in both waves. This longitudinal sample is restricted to those adolescents young enough in the 2017-18 wave (ages 10–13) to still be aged 10–15 in November 2020 (and thus fall within the age restriction of the COVID-19 adolescent survey). *N* = 20 are excluded due to missing information, resulting in an analytical sample of *n* = 1,214 person-observations. Inverse probability weights are constructed to reduce bias from attrition, correcting for any differential attrition between 2017 and 18 and November 2020 by emotional-behavioral difficulties, community disadvantage, adolescents’ age, gender, ethnicity and household-level family structure, financial situation, employment status, and education. This inverse probability weight is then multiplied by the 2017-18 wave cross-sectional weight (see Supplementary-Appendix: S.2 for descriptives of the longitudinal panel sample). Both listwise deletion and multiple imputation using chained equations (20 imputed datasets) were conducted to assess the impact of within-case missing data (while full case missingness from attrition is adjusted for via inverse probability weighting, as above). Given that within-case missingness was minimal and both approaches yielded substantively identical results, listwise deletion results are reported for simplicity.

Special license access versions of both datasets are used which provide information on the Local Authority Districts (LADs; average population: ~160,000 residents) respondents were living in. LAD-level COVID-19 case data is based on UK government data. Contextual social capital is measured using the UK Social Fabric Index (Tanner et al., 2020). LAD-level disadvantage is measured using UK Census data. 

### Pandemic Context

At the three peri-pandemic time points, adolescents experienced various degrees of pandemic-related school disruption and social restrictions. The UK entered a full national lockdown on 23rd March 2020, with schools closing. A phased ending of the lockdown began mid-June, and although some schools reopened, most students remained at home up until the summer holidays. Therefore, by August 2020 (wave 1 of the COVID-19 survey), most adolescents had been out of school for 4–5 months. Schools reopened at the start of the academic year in September 2020 and remained open to November-December 2020 (wave 2 of the COVID-19 survey), although mandates restricting wider social mixing were in place, including a second national lockdown (November 2020 onwards). In March 2021 (wave 3 of the COVID-19 survey), schools had been closed from the end of December until 8th March (third full UK lockdown), after which staggered reopening began, although wider mixing restrictions (e.g., stay-at-home orders) remained in place until 29th March.

### Internalizing and Externalizing Difficulties

Adolescents’ emotional and behavioral problems, defined as difficulties in regulating emotions and behavior that interfere with daily functioning and social relationships, were assessed using the adolescent self-report version of the Strengths and Difficulties Questionnaire (SDQ) (Goodman et al., 2003). The SDQ is a widely used behavioral screening tool designed to identify emotional and behavioral problems in children and adolescents. Two subdimensions were analyzed. The externalizing score comprises seven items (e.g., “I get very angry and often lose my temper”; “I am restless, I cannot stay still for long”) that assess conduct problems and hyperactivity/inattention. The internalizing score also includes seven items (e.g., “I worry a lot”; “I am often unhappy, downhearted, or tearful”) capturing emotional symptoms and peer relationship problems. Each subscale ranges from 0 to 20, with higher scores indicating greater difficulties. In the present study, internal consistencies were acceptable (Alpha scores: internalizing 0.88 and externalizing 0.87), consistent with previous research demonstrating reliability and validity of the SDQ across adolescent samples (Goodman et al., 2003).

### Family Social Capital: Eating Meals Together

The family dimension of adolescents’ strong tie social capital is measured by how frequently an adolescent eats evening meals with their family (previously applied to capture the strength of family networks, e.g., Rothon et al., 2012). Adolescents were asked: “In the past 7 days, how many times have you eaten an evening meal together with the rest of your family who live with you?”. Responses ranged from “None” (1), “1–2 times” (2), “3–5 times” (3), and “6–7 times” (4). This measure was available in the 2017-18 wave of the Mainstage survey and in the November 2020 wave of the COVID-19 Study.

### Friend Social Capital: Number of Close Friends

The friendship dimension of adolescents’ strong tie social capital is measured via their reported number of close friends: “How many close friends do you have, people that you feel at ease with, can talk to about private matters?” (count variable). This measure was available in the 2017-18 wave of the Mainstage survey and in the November 2020 wave of the COVID-19 Study.

### Extra-curricular Involvement

Adolescent involvement in extracurricular activities is measured via the question: “What activities do you usually do outside of lesson time other than those involving physical activity?” Activities included “Youth club”, “Scouts/Guides”, “Music classes”, “Cultural groups”, “Religious groups”, and “Other activities”. A count variable (0–6) is calculated by summing the number of activities an adolescent was involved in. This was measured in the November 2020 wave of the COVID-19 Study.

### Neighborhood Social Capital

#### Adolescent perceived neighborhood safety

Adolescents’ self-reported neighborhood social capital is measured via their perceptions about others in their neighborhood, in particular, how safe they feel in their neighborhood (Duke et al., 2009). Respondents were asked: “How safe would you feel walking alone in your neighborhood after dark?” Responses included “Very unsafe” (1), “A bit unsafe” (2), “Fairly safe” (3), and “Very safe” (4). This measure was available in the 2017-18 wave of the Mainstage survey and in the November 2020 wave of the COVID-19 Study.

#### Household-level neighborhood social capital

Parents’ social embeddedness in their neighborhoods (household social capital) is captured by averaging at the household-level parents’ reported neighborhood social capital. This is a mean score (ranging from 1 to 5) of two social capital indicators reported by parents in the household: “I regularly stop and talk with people in my neighborhood” and “I think of myself as similar to the people that live in this neighborhood”. Reponses included “Strongly disagree” (1), “Disagree” (2), “Neither agree nor disagree” (3), “Agree” (4), and “Strongly agree” (5). This measure was available in the November 2020 wave of the COVID-19 Study.

#### Community-level (Local Authority) neighborhood social capital

The contextual-level of social capital in adolescents’ wider community is measured using the Local Relationships dimension of the 2018-19 UK Social Fabric Index. This indicator captures the pre-pandemic (2019) degree of neighborliness and associational involvement in a Local Authority (Tanner et al. 2020). This index is composed of administrative data sources, e.g., per capita rates of community-owned shops or pubs, amateur sports clubs, membership organizations, or residents’ associations, alongside aggregated indicators of neighborliness from the UKHLS Mainstage data, e.g., share of people who participate in a local organization, share of people who agree that “this is a close-knit neighborhood”. Scores on this index range from 3 to 6.5.

### Perceived Family Social Support

Perceived family social support is measured by the question: “Do you feel supported by your family, that is the people who live with you?” Responses include “I do not feel supported by my family in the things I do” (1), “I feel supported by my family in some of the things I do” (2), and “I feel supported by my family in most or all of the things I do” (3). This measure was available in the 2017-18 wave of the Mainstage survey and in the November 2020 wave of the COVID-19 Study.

### Perceived Friend Social Support

Perceived friend social support is measured by the question: “Do you feel supported by your friends?” Responses include “I do not feel supported by my friends at all” (1), “I feel supported by my friends some of the time” (2), and “I feel supported by my friends most of the time” (3). This was measured in the November 2020 wave of the COVID-19 Study. Testing using factor analysis showed that indicators of family and friend social support do not load on to a single, shared dimension of social support, suggesting they pick up distinct sources.

### Age

Adolescents were grouped into three age categories: ages 10–11, 12–13 and 14–15.

### Gender

Adolescent gender was coded as male and female.

### Ethnicity

Adolescents were grouped based on their self-reported ethnicity into White, Mixed ethnicity, Asian, Black and Other ethnic group.

### Disability Status

Parents reported whether the child had a health condition which limits him/her at play or from joining in any other activity normal for a child his/her age. Categories include ‘no health condition’, ‘limited a little’ or ‘limited a lot’.

### Household Education Status

The qualification status of households is captured by comparing households where neither parent has a ‘degree level qualification or higher’ and households where at least one parent has a ‘degree level qualification or higher’.

### Household Employment Status

The employment status of a household is captured by measuring the number of people employed in the household.

### Household Subjective Financial Situation

The subjective financial situation of households is calculated by taking the average response of caregivers present in the household to the question: “How well would you say you yourself are managing financially these days? Would you say you are…” “Finding it very difficult” (1) to “Living comfortably” (5). Mean household scores of < 2.5 were coded as “finding it difficult” and scores of ≥ 2.5 were coded as “living comfortably”.

### Household Lone Parent Status

Households were coded by whether they contain one parent or whether there was a partner in the household.

### Community (Local Authority) Level socio-economic Disadvantage

Two indices of socio-economic disadvantage are constructed using factor analysis of the characteristics of the Local Authority in which adolescents lived: an index of economic disadvantage (% social housing, % unemployed, % female lone-parent households) and social disadvantage (% without degrees, % not in managerial and professional roles) from UK Census data (factor loadings > 0.4; Eigen values > 1; Alpha scores > 0.7).

### Community (Local Authority) Level COVID-19 Intensity

To capture the intensity of the pandemic in adolescents’ communities’ models include COVID-19 case rate per 1,000 at the time of the survey.

### Analytic Approach

The key aim of this study is to explore whether adolescents’ peri-pandemic social capital cushioned (that is, moderated) their pre- to peri-pandemic trajectories in SDQ scores over the COVID-19 pandemic. Two approaches are taken (see Table 1 for full details). The first involves tracking adolescents’ SDQ scores across all 4-waves of data: 1 pre-pandemic wave and 3 peri-pandemic waves. It then tests whether these SDQ trends differ across levels of peri-pandemic social capital reported by adolescents in November 2020 (when social capital measures were taken). Adolescent social capital is therefore fixed at its November 2020 value across all pre-/peri-pandemic waves of data. To model these trends, the data are treated as pooled cross-sections, pooling all four waves, and analyzed using 3-level multi-level mixed regression models (level-1: observations; level-2: adolescents; level-3: Local Authorities), alongside survey-wave dummy variables (to track trends in SDQ), with random-coefficients for survey-wave included at the Local Authority level. To test whether social capital buffered the impact of the pandemic on SDQ, interaction-terms between social capital indicators and each survey-wave are included (with the pre-pandemic survey wave excluded as baseline). A significant, negative interaction-term signifies that individuals with higher peri-pandemic social capital experienced smaller pre- to peri-pandemic changes in emotional-behavioral difficulties (evidence of stress-buffering). Thus, the difference (gap) in SDQ scores between individuals with higher/lower social capital has significantly widened (relative to the pre-pandemic period).

Models are built-up in stages. Model 1 tests the pre- to peri-pandemic time trend in SDQ (survey-period dummies), adjusting for covariates. Model 2 adds in all measures of peri-pandemic social capital to test their direct associations with SDQ. Models 3–6 then focus on testing the posited moderating role of peri-pandemic social capital. Model 3 analyses family/friend strong-tie connectivity, adding interaction-terms between survey-period and number of close friends and frequency of family meals to the model. Model 4 then removes measures of friend/family social capital and includes extra-curricular involvement, neighborhood safety (neighborhood connection), and measures of household- and contextual-level social capital (alongside their attendant survey-period interaction-terms). Model 5 then includes all social capital indicators and attendant survey-period interaction-terms in a single model to test their independence when modelled together. Model 6 then adds in measures of perceived friend and family social support and their attendant survey-period interaction-terms. This will test whether perceived social support also moderated trends in SDQ over the pandemic and whether any observed moderating association by adolescent social capital is reduced when accounting for the moderating role of perceived social support, i.e., whether any apparent buffering effect of social capital can be explained by adolescents’ social support. Separate analyses are conducted for SDQ internalizing and externalizing scores.

This first analytic approach has several advantages. It allows us to track differences in pre-/peri-pandemic trends across all four waves of data. This approach can also explore the potential stress-buffering role of dimensions of peri-pandemic social capital only measured in November 2020, e.g., friend social support, usual extra-curricular involvement (see Table 1). The second analytic approach then aims to more robustly test any stress-buffering role of social capital identified in the first approach. To do so, the data are treated as a longitudinal panel dataset, employing individual-level fixed-effects modelling to account for potential bias from time-invariant unobserved heterogeneity. These models only utilize the 2017-18 wave (pre-pandemic) and November 2020 wave (peri-pandemic) of data (the only waves for which there are repeat measures of SDQ/social capital). Although fewer social capital measures can be tested on fewer waves of data, these models undertake a more causally robust analysis. Table 1 summarizes the data structure of the two analytic approaches. Sensitivity/robustness testing is undertaken throughout. Social capital measures are mean centered to reduce potential bias from multicollinearity.

**Table 1:** Summary of Analytic Approaches

			Wave of UKHLS data			
Analytical approach	Treatment of data	Measure	2017-18	August 2020	November 2020	March 2021
			Pre-pandemic		Peri-pandemic	
1 (multilevel mixed)	Pooled cross-sectional	SDQ	At wave value	At wave value	At wave value	At wave value
		Social capital	Fixed at Nov. 2020 value	Fixed at Nov. 2020 value	Fixed at Nov. 2020 value	Fixed at Nov. 2020 value
		Covariates	At wave value	At wave value	At wave value	At wave value
2 (individual fixed effects)	Longitudinal panel	SDQ	At wave value		At wave value	
		Social capital	At wave value		At wave value	
		Covariates	At wave value		At wave value	

*Notes*: UKHLS = United Kingdom Household Longitudinal Study; SDQ = Strengths and Difficulties Questionnaire

## Results

### Preliminary Results

Table 2 examines the correlations between the key study variables (SDQ scores across all four waves and peri-pandemic social capital fixed at its November 2020 value). SDQ internalizing and externalizing scores are moderately correlated. On the whole, dimensions of social capital are negatively related to symptoms of adolescent internalizing/externalizing difficulties, although they are more closely associated with internalizing scores than externalizing scores. Dimensions of stronger-tie connectivity (friend/family network structure) are also more closely associated with adolescents’ SDQ, while dimensions of weaker-tie connectivity (neighborhood safety, extra-curricular involvement, parental-/community-level contextual social capital) tend to have low associations with SDQ scores. Neighborhood safety is somewhat of an exception, being more strongly correlated with SDQ (especially internalizing) scores than other dimensions of weaker-tie connectivity. Perceived friend and family support, however, have the strongest negative association with SDQ scores.

**Table 2:** Bivariate Correlations between Key Study Variables

	1.	2.	3.	4.	5.	6.	7.	8.	9.	10.
1. SDQ Internalizing	1									
2. SDQ Externalizing	0.44***	1								
*Social capital (Nov 2020)*										
3. Eating family meal (F)	-0.14***	-0.16***	1							
4. Close friends (N)	-0.16***	-0.03	0.04*	1						
5. Family support	-0.36***	-0.29***	0.24***	0.09***	1					
6. Friend support	-0.33***	-0.21***	0.16***	0.22***	0.37***	1				
7. Neigh. safety	-0.24***	-0.08***	0.05**	0.12***	0.16***	0.13***	1			
8. HH social capital	-0.02	-0.02	0.03	0.03+	0.05**	0.02	0.06**	1		
9. LA Relationships Index	-0.05**	-0.01	-0.02	0.04*	-0.05**	0.02	0.08***	0.06***	1	
10. Extra-curricular (N)	-0.01	-0.04*	0.02	0.05**	-0.01	0.05**	0.04*	0.02	0.05**	1

F = Frequency; N = Number; HH = Household; LA = Local Authority; SDQ = Strengths and Difficulties Questionnaire

+ *p* < .10, * *p* < .05, ** *p* < .01, *** *p* < .001

### Internalizing Difficulties

The first analytic stage explores whether trends in SDQ scores (across all four waves) are buffered (moderated) by adolescents’ peri-pandemic social capital. Table 3 examines internalizing scores (models contain all individual-, household- and community-level covariates although not shown - see Supplementary-Appendix: S.3 for full results). Model 1 (Table 3) demonstrates that adolescent internalizing scores increased significantly with the onset of the pandemic (2017-18 to August 2020) and remained persistently elevated into March 2021 (around 0.5 points above their pre-pandemic level). Model 2 includes all measures of peri-pandemic social capital to first establish their overall associations with internalizing scores, demonstrating that number of close friends, frequency of family meals, and higher neighborhood safety are independently associated with fewer internalizing symptoms.

**Table 3:** Multilevel Mixed Regressions Examining Trends in SDQ Internalizing Scores across Levels of Adolescent Social Capital (Pooled Cross-sectional Approach)

	Model 1	Model 2	Model 3	Model 4	Model 5	Model 6
SDQ type	Internalize	Internalize	Internalize	Internalize	Internalize	Internalize
baseline - Survey wave 2017-18	ref.	ref.	ref.	ref.	ref.	ref.
Aug-20	0.491*	0.405+	2.341***	1.554	2.722*	5.717***
	(0.223)	(0.221)	(0.677)	(1.238)	(1.334)	(1.427)
Nov-20	0.570**	0.482*	3.018***	2.101	3.648**	8.708***
	(0.218)	(0.220)	(0.721)	(1.389)	(1.361)	(1.587)
Mar-21	0.575**	0.492*	3.409***	1.945	4.095**	7.117***
	(0.218)	(0.216)	(0.721)	(1.388)	(1.345)	(1.522)
*Social capital measures*						
Eating family meal (F)		-0.392***	-0.058		-0.065	0.031
		(0.111)	(0.179)		(0.177)	(0.177)
August 2020 * Family meal (F)			-0.311		-0.301	-0.136
			(0.192)		(0.194)	(0.204)
November 2020 * Family meal (F)			-0.493*		-0.468*	-0.206
			(0.203)		(0.207)	(0.201)
March 2021 * Family meal (F)			-0.594**		-0.570**	-0.461*
			(0.199)		(0.199)	(0.203)
Close friends (N)		-0.105***	-0.031		-0.026	-0.010
		(0.019)	(0.040)		(0.039)	(0.040)
August 2020 * Close friends (N)			-0.123**		-0.113**	-0.094*
			(0.039)		(0.038)	(0.039)
November 2020 * Close friends (N)			-0.117**		-0.104*	-0.082+
			(0.044)		(0.043)	(0.042)
March 2021 * Close friends (N)			-0.122**		-0.110**	-0.092*
			(0.044)		(0.042)	(0.044)
Neighborhood safety		-0.742***		-0.508*	-0.532**	-0.459*
		(0.119)		(0.208)	(0.200)	(0.205)
August 2020 * Neigh. safety				-0.377+	-0.231	-0.150
				(0.215)	(0.209)	(0.215)
November 2020 * Neigh. safety				-0.470*	-0.305	-0.166
				(0.229)	(0.221)	(0.215)
March 2021 * Neigh. safety				-0.462*	-0.265	-0.190
				(0.236)	(0.237)	(0.247)
HH social capital		-0.041		-0.033	-0.034	-0.052
		(0.086)		(0.096)	(0.095)	(0.092)
August 2020 * HH Social capital				-0.054	-0.036	0.037
				(0.221)	(0.217)	(0.214)
November 2020 * HH Social capital				-0.007	0.023	0.120
				(0.247)	(0.250)	(0.224)
March 2021 * HH Social capital				-0.012	-0.029	-0.000
				(0.228)	(0.232)	(0.227)
LA Relationships Index		-0.093		-0.042	-0.074	-0.164
		(0.210)		(0.272)	(0.271)	(0.267)
August 2020 * LA Relations. Index				0.006	0.035	0.010
				(0.224)	(0.222)	(0.224)
November 2020 * LA Relations. Index				-0.087	-0.041	-0.101
				(0.220)	(0.209)	(0.207)
March 2021 * LA Relations. Index				-0.056	-0.025	-0.022
				(0.200)	(0.189)	(0.190)
Extra-curricular involvement (N)		0.194		0.279	0.270	0.274
		(0.107)		(0.192)	(0.185)	(0.183)
August 2020 * Extra-curricular (N)				-0.067	-0.001	-0.008
				(0.206)	(0.202)	(0.203)
November 2020 * Extra-curricular (N)				-0.178	-0.116	-0.112
				(0.215)	(0.209)	(0.207)
March 2021 *Extra-curricular (N)				-0.218	-0.146	-0.153
				(0.222)	(0.215)	(0.221)
Family support						-0.747*
						(0.360)
August 2020 * Family support						-0.801*
						(0.403)
November 2020 * Family support						-1.414***
						(0.396)
March 2021 * Family support						-0.426
						(0.483)
Friend support						-0.492
						(0.330)
August 2020 * Friend support						-0.670*
						(0.338)
November 2020 * Friend support						-0.969**
						(0.345)
March 2021 * Friend support						-0.954**
						(0.376)
Constant	3.858***	8.409***	4.227***	5.542***	6.083***	9.060***
	(0.352)	(0.972)	(0.719)	(1.208)	(1.302)	(1.488)
AIC	14972.75	14874.67	14873.32	14930.85	14850.31	14652.26
BIC	15160.71	15099.01	15109.79	15215.83	15183.79	15034.25
Observations	3176	3176	3176	3176	3176	3176

F = Frequency; N = Number; HH = Household; LA = Local Authority; Relations. = Relationships; Neigh. = Neighbourhood; SDQ = Strengths and Difficulties Questionnaire; Internalize = Internalizing scores; UK Household Longitudinal Study Mainstage and UK Household Longitudinal Study COVID-19 data; standard errors in parentheses; models contain all covariates (full results in Supplementary-Appendix: S.3)

*+ p <* .10, * p < .05, ** p < .01, *** p < .001

Models 3–5 (Table 3) explore whether peri-pandemic social capital moderated trends in internalizing SDQ via testing interaction-terms between each survey-period and different forms of social capital. Where a significant moderating relationship is identified in the models, trends in predicted internalizing scores (derived from the models) across each survey-period are plotted but subdivided by whether adolescents reported high/low levels of peri-pandemic social capital to investigate the substantive implications of these relationships. Statistical significance markers on the figures located above each peri-pandemic survey wave (August 2020; November 2020; March 2021) indicate where a difference in internalizing scores between the high/low social capital groups is significantly different from the difference in SDQ scores in the 2017-18 baseline wave (reflective of results reported in Table 3), i.e., evidence of significant variation in trends in internalizing scores across levels of social capital.

**Fig. 1:**
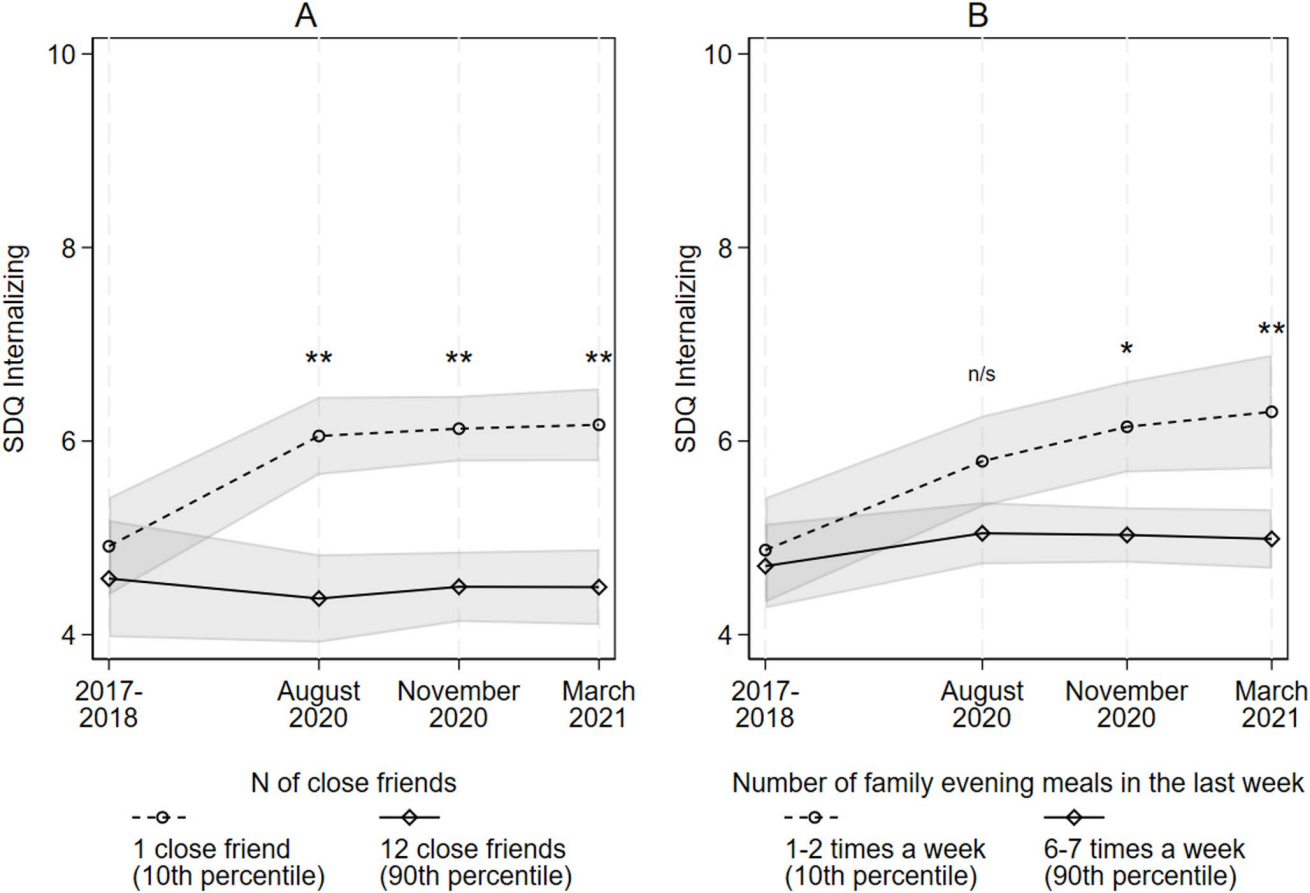
Trends in SDQ Internalizing by adolescents’ peri-pandemic number of close friends (A) and frequency of family meals (B). *Notes: *shaded areas represent 95% confidence intervals; results based on Model 3, Table 3; significance markers indicate where a difference in scores between high/low social capital groups is significantly different from the difference in scores in 2017-18; SDQ = Strengths and Difficulties Questionnaire. + p < .10, * p < .05, ** p < .01, *** p < .001

Figure 1 (Panels A and B) demonstrate how the number of close friends reported by adolescents and their frequency of family meals during the pandemic (indicators of strong tie social capital) significantly moderated pre- to peri-pandemic trends in internalizing difficulties (based on Model 3, Table 3). Adolescents in the 10th percentile of close friends experienced a significant pre-/peri-pandemic increase in internalizing scores (+ 1.12 [0.54, 1.7]), which remained elevated across the pandemic (Fig. 1, Panel A). However, adolescents in the 90th percentile experienced no significant pre- to peri-pandemic change in scores (-0.24 [-0.85, 0.38]), which remained stable and low over the pandemic. Adolescents who only ate meals with their family 1–2 times a week (low social capital) also saw a significant pre- to peri-pandemic (August 2020) increase in internalizing scores (+ 0.91 [0.3, 1.52]) while those who ate meals 6–7 times a week (high) saw no significant change (+ 0.16 [-0.44, 0.76]) (Fig. 1, Panel B). The difference between these initial trends is marginally non-significant at the *p* < .1 level. However, internalizing scores continued to increase among those only eating family meals 1–2 times a week (an additional 0.5 points by March 2021) while scores remained stable and low for the high family meal frequency group. Accordingly, by November 2020 and into March 2021, internalizing scores had increased significantly more among the low frequency group (relative to the pre-pandemic period), suggesting a significant moderating role of family networks emerged over the pandemic.

**Fig. 2:**
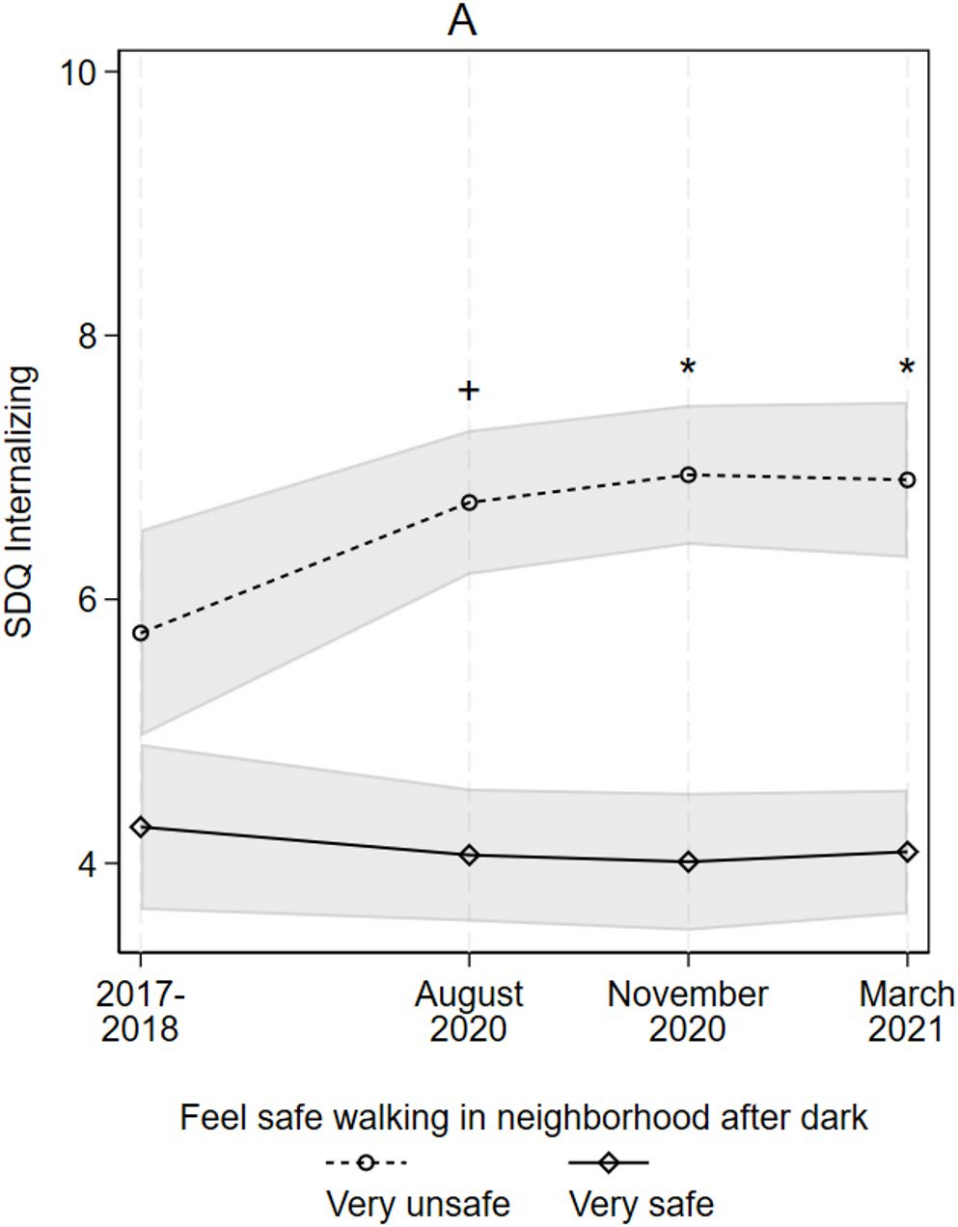
Trends in SDQ Internalizing by adolescents’ peri-pandemic neighborhood safety (A). *Notes*: shaded areas represent 95% confidence intervals; results based on Model 5, Table 3; significance markers indicate where a difference in scores between high/low social capital groups is significantly different from the difference in scores in 2017-18; SDQ = Strengths and Difficulties Questionnaire. + p < .10, * p < .05, ** p < .01, *** p < .001

To examine the role of extra-curricular activity and indicators of neighborhood social capital, Model 4 (Table 3) removes all measures of friend/family social capital and explores the moderating roles of extra-curricular involvement, perceived neighborhood safety, parents’ reported neighborhood social capital, and contextual-level community social capital. None of the interaction-terms between survey-period and extra-curricular involvement, parent reported social capital, or contextual social capital are statistically significant. However, Fig. 2 (Panel A) demonstrates how trends in internalizing scores are moderated by how safe adolescents felt in their neighborhood (based on Model 4, Table 3). Adolescents who felt very unsafe during the pandemic saw a significant pre- to peri-pandemic (August 2020) increase in internalizing scores (0.94 points [0.07, 1.81]), after which scores remained elevated. Adolescents who felt very safe saw no significant change in internalizing scores with the onset of the pandemic (-0.22 points [-0.89, 0.45]), which remained stable and comparatively low.

Model 5 tests the independence of different dimensions of adolescents’ social capital by including all measures of social capital and their attendant survey-period interaction-terms simultaneously in a model. While the moderating roles of friend/family social capital remain broadly unchanged, the interaction-terms between survey-period and adolescents’ perceived neighborhood safety are reduced in size and rendered non-significant. Any apparent stress-buffering role of perceived safety thus appears to be driven by adolescents who feel safer also reporting greater friend and family social capital.

**Fig. 3:**
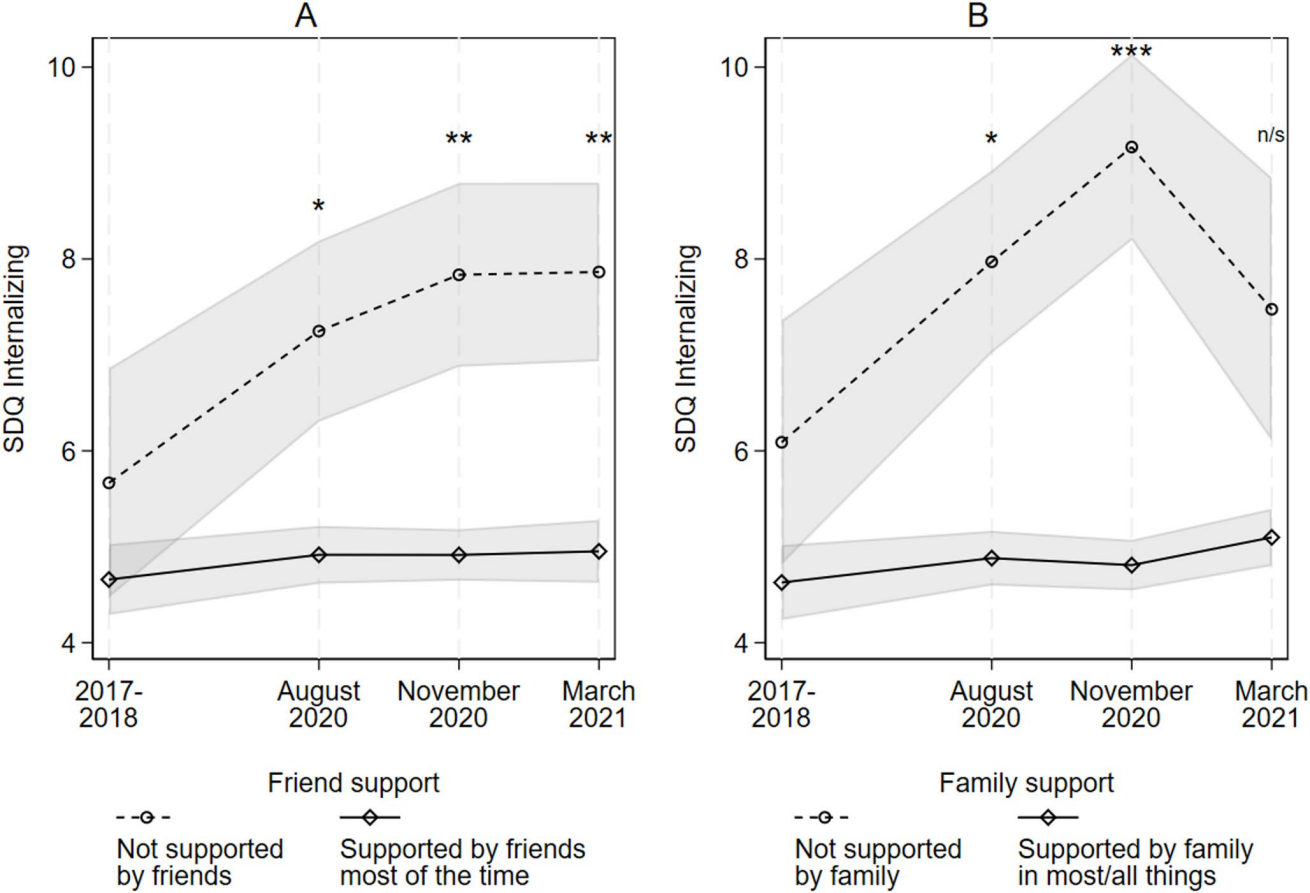
Trends in SDQ Internalizing by adolescents’ peri-pandemic social support from friends (A) and family (B). *Notes*: shaded areas represent 95% confidence intervals; results based on Model 4, Table 3; significance markers indicate where a difference in scores between high/low social capital groups is significantly different from the difference in scores in 2017-18; SDQ = Strengths and Difficulties Questionnaire. + p < .10, * p < .05, ** p < .01, *** p < .001

To explore the posited role of perceived social support in explaining why social capital might buffer the impact of the pandemic (i.e., the stress-buffering model), Model 6 adds measures of adolescents’ peri-pandemic perceived friend and family social support, and their attendant survey-period interaction-terms. Figure 3 (Panels A and B) demonstrates how the adolescents’ peri-pandemic friend and family social support significantly moderated trends in internalizing scores over the pandemic (based on Model 6, Table 3). Adolescents who reported having no support from friends saw a significant 1.74 [0.5, 2.98] point increase in internalizing SDQ over the pre-/peri-pandemic (August 2020) period, which then slowed and stabilized into March 2021 (Fig. 3, Panel A). Adolescents who felt supported by their friends most of the time, however, saw no significant change (+ 0.34 [-0.09, 0.77] points), with scores remaining comparatively low and stable. Similarly, adolescents who reported no peri-pandemic support from their family experienced a significant 2.01 [0.61, 3.42] point increase in internalizing symptoms over the pre-/peri-pandemic (August 2020) period, which increased again into November 2020 (+ 1.11 [0.13, 2.08]) (Fig. 3, Panel B). However, their internalizing scores then significantly declined by 1.63 [-2.78, − 0.48] points into March 2021. Meanwhile, adolescents reporting high family support saw no significant change in scores over the pandemic. Peri-pandemic family support therefore moderated trends in internalizing SDQ in the early to middle period of the pandemic, but this association had weakened and was no longer significant by March 2021 (even though internalizing scores remained elevated for the low-family support group).

After accounting for the moderating role of perceived social support, the interaction-terms between survey-period and family social capital are reduced in statistical significance and size (between 19 and 56 per cent) (comparing Model 5 to Model 6). The interaction-terms between survey-period and close friend social capital are also reduced in statistical significance and size, although by less than for family social capital (between 16 and 21 per cent). This suggests that a significant part of why adolescents who report greater family/friend social capital exhibit smaller increases in internalizing scores over the pandemic is that they have higher levels of perceived friend/family social support. However, of note, is that family, and especially close friend, social capital continues to independently moderate trends in internalizing scores even after accounting for their levels of perceived social support, suggesting other mediating pathways are in operation. Model fit statistics (AIC; BIC) were used to examine model fit and parsimony. Examining fit following the addition of social capital measures (Models 1–5), the inclusion of stronger-tie dimensions of social capital (and period-interactions) (Model 3) was associated with the largest improvements in model fit (AIC). However, ultimately, Model 6 (including all dimensions of social capital and social support) exhibited the most optimal fit as well as the lowest BIC.

### Externalizing Difficulties

The next analytic stage examines the stress-buffering role of social capital for SDQ externalizing scores (Table 4). Models contain all individual-, household- and community-level covariates although not shown (see Supplementary-Appendix: S.4 for full results). Model 1 (Table 4) demonstrates that adolescent externalizing scores reacted somewhat differently to internalizing scores. Between 2017 and 18 and August 2020, adolescents saw a small but non-significant increase. However, scores then increased into November 2020 (a 0.84-point increase on their pre-pandemic value) before dropping again into March 2021, although remaining significantly elevated. Model 2 includes all peri-pandemic social capital measures, demonstrating how adolescents who ate family meals more frequently and felt safer in their neighborhood during the pandemic reported fewer externalizing symptoms.

**Table 4:** Multilevel Mixed Regressions Examining Trends in SDQ Externalizing Scores across Levels of Adolescent Social Capital (Pooled Cross-Sectional Approach)

	Model 1	Model 2	Model 3	Model 4	Model 5	Model 6
SDQ type	Externalize	Externalize	Externalize	Externalize	Externalize	Externalize
baseline - Survey wave 2017-18	ref.	ref.	ref.	ref.	ref.	ref.
Aug-20	0.254	0.248	0.645	1.836+	1.880	0.963
	(0.206)	(0.205)	(0.837)	(1.069)	(1.285)	(1.505)
Nov-20	0.857**	0.862**	1.630*	2.191+	2.633+	4.333*
	(0.289)	(0.287)	(0.694)	(1.299)	(1.388)	(1.683)
Mar-21	0.453*	0.458*	2.075**	2.161+	3.527**	4.909**
	(0.219)	(0.217)	(0.764)	(1.217)	(1.363)	(1.641)
Social capital measures						
Eating family meal (F)		-0.549***	-0.411*		-0.433**	-0.258
		(0.112)	(0.169)		(0.167)	(0.174)
August 2020 * Family meal (F)			-0.035		0.002	-0.079
			(0.225)		(0.222)	(0.227)
November 2020 * Family meal (F)			-0.179		-0.137	-0.031
			(0.193)		(0.191)	(0.191)
March 2021 * Family meal (F)			-0.410+		-0.367+	-0.297
			(0.211)		(0.209)	(0.209)
Close friends (N)		-0.010	0.002		-0.002	0.019
		(0.017)	(0.032)		(0.032)	(0.032)
August 2020 * Close friends (N)			-0.040		-0.021	-0.025
			(0.035)		(0.035)	(0.036)
November 2020 * Close friends (N)			-0.019		-0.004	0.001
			(0.042)		(0.040)	(0.040)
March 2021 * Close friends (N)			-0.027		-0.009	-0.004
			(0.040)		(0.040)	(0.039)
Neighborhood safety		-0.355**		0.011	0.009	0.120
		(0.114)		(0.167)	(0.167)	(0.164)
August 2020 * Neigh. safety				-0.487**	-0.455*	-0.523**
				(0.186)	(0.187)	(0.188)
November 2020 * Neigh. safety				-0.421*	-0.397*	-0.337+
				(0.193)	(0.185)	(0.183)
March 2021 * Neigh. safety				-0.599***	-0.556**	-0.502**
				(0.190)	(0.190)	(0.195)
HH social capital		-0.024		0.049	0.061	0.053
		(0.100)		(0.112)	(0.114)	(0.112)
August 2020 * HH Social capital				-0.351+	-0.350+	-0.357+
				(0.190)	(0.190)	(0.194)
November 2020 * HH Social capital				-0.188	-0.183	-0.112
				(0.227)	(0.227)	(0.227)
March 2021 * HH Social capital				-0.194	-0.223	-0.200
				(0.213)	(0.214)	(0.215)
LA Relationships Index		-0.137		-0.207	-0.246	-0.341
		(0.241)		(0.284)	(0.281)	(0.277)
August 2020 * LA Relations. Index				0.261	0.265	0.335+
				(0.188)	(0.186)	(0.187)
November 2020 * LA Relations. Index				0.146	0.145	0.172
				(0.169)	(0.166)	(0.161)
March 2021 * LA Relations. Index				0.158	0.149	0.166
				(0.179)	(0.177)	(0.175)
Extra-curricular involvement (N)		-0.007		0.192	0.189	0.185
		(0.113)		(0.158)	(0.157)	(0.159)
August 2020 * Extra-curricular (N)				-0.196	-0.177	-0.153
				(0.183)	(0.181)	(0.174)
November 2020 * Extra-curricular (N)				-0.358*	-0.337*	-0.337*
				(0.166)	(0.165)	(0.165)
March 2021 *Extra-curricular (N)				-0.201	-0.167	-0.195
				(0.203)	(0.194)	(0.194)
Family support						-1.112**
						(0.377)
August 2020 * Family support						0.613
						(0.498)
November 2020 * Family support						-0.714
						(0.480)
March 2021 * Family support						-0.541
						(0.520)
Friend support						-0.608*
						(0.302)
August 2020 * Friend support						-0.185
						(0.394)
November 2020 * Friend support						-0.288
						(0.365)
March 2021 * Friend support						-0.161
						(0.391)
Constant	5.429***	8.923***	6.781***	5.999***	7.558***	11.508***
	(0.371)	(1.129)	(0.696)	(1.291)	(1.380)	(1.611)
AIC	14964.08	14940.52	14940.95	14954.04	14935.43	14834.01
BIC	15152.04	15164.87	15177.42	15239.02	15268.91	15216.01
Observations	3176	3176	3176	3176	3176	3176

F = Frequency; N = Number; HH = Household; LA = Local Authority; Relations. = Relationships; Neigh. = Neighbourhood; SDQ = Strengths and Difficulties Questionnaire; Externalize = Externalizing scores; UK Household Longitudinal Study Mainstage and UK Household Longitudinal Study COVID-19 data; standard errors in parentheses; models contain all covariates (full results in Supplementary-Appendix: S.4)

+ p < .10, * p < .05, ** p < .01, *** p < .001

Models 3–5 (Table 4) explore whether peri-pandemic social capital moderated trends in externalizing scores via testing interaction-terms between survey-periods and different dimensions of social capital. Unlike for internalizing scores, there is little consistent evidence that family/friend network structure (Model 3) moderated trends in externalizing scores. In addition, neither contextual-level community social capital nor parent-reported neighborhood social capital consistently moderated trends in externalizing scores (Model 4). However, the results do show that perceived neighborhood safety and, to a lesser extent, extracurricular involvement appeared to moderate trends in externalizing scores over the pandemic.

**Fig. 4:**
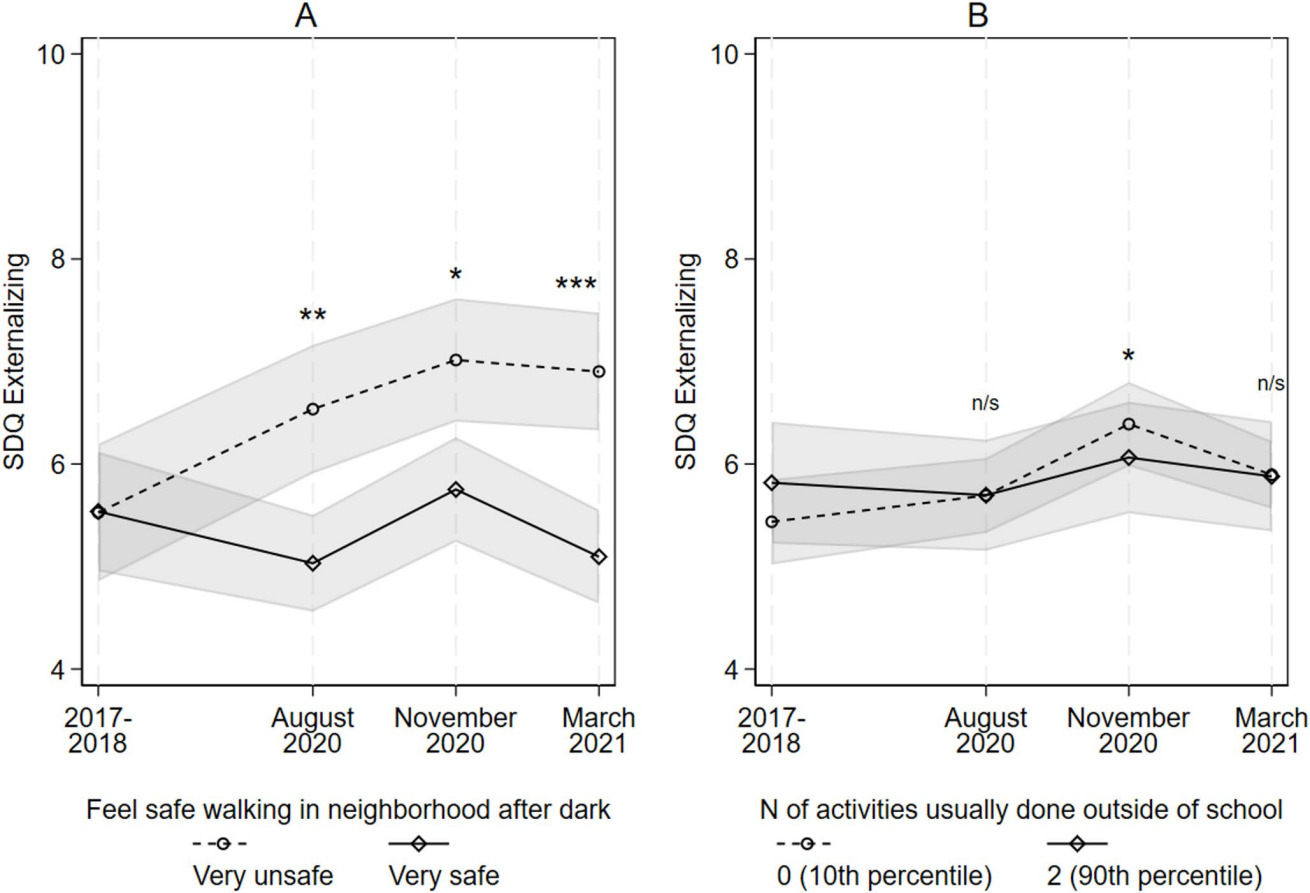
Trends in SDQ Externalizing by adolescents’ peri-pandemic neighborhood safety (A) and usual extra-curricular activity (B). Notes: shaded areas represent 95% confidence intervals; results based on Model 5, Table 3; significance markers indicate where a difference in scores between high/low social capital groups is significantly different from the difference in scores in 2017-18; SDQ = Strengths and Difficulties Questionnaire. + p < .10, * p < .05, ** p < .01, *** p < .001

Figure 4 (Panel A) demonstrates how neighborhood safety moderated trends in externalizing scores (based on Model 4, Table 4). Adolescents who felt unsafe in their neighborhood saw a significant 0.99 [0.26, 1.73] point increase in externalizing scores over the pre- to peri-pandemic (August 2020) period, which then broadly stabilized over the pandemic. Adolescents feeling safe saw greater fluctuation in their scores, exhibiting no significant change (-0.54 points [-1.14, 0.07]) at onset of the pandemic, before subsequent increases and declines. However, over the course of the pandemic, neighborhood safety remained a significant moderator of externalizing SDQ. There is also some weaker evidence that trends in externalizing problems were moderated by involvement in extra-curricular activities (Model 5). Figure 4 (Panel B) demonstrates no difference in changes in pre- to peri-pandemic (August 2020) externalizing scores based on how much adolescents were usually involved in extra-curricular activities (based on Model 5, Table 4). Adolescents in the 10th percentile of involvement did see a significant increase of 0.7-points [0.17, 1.23] between August 2020 and November 2020, while scores among those in the 90th percentile of involvement saw no change. However, scores among the low-involvement group then declined again into March 2021. These two moderating relationships remain statistically significant even when modelled alongside strong tie friend/family social capital (and their attendant survey-period interaction terms) (Model 5).

Lastly, the analysis examines whether perceived social support can account for any apparent buffering role of social capital for trends in externalizing scores. Model 6 (Table 4) adds measures of adolescents’ peri-pandemic perceived friend and family social support alongside their attendant survey-period interaction terms. However, there is no evidence that perceived social support moderated trends in externalizing scores. In addition, the interaction-terms between survey-period and either perceived neighborhood safety or extracurricular involvement remain broadly unchanged.

Model fit statistics (AIC; BIC) assess models for fit and parsimony. The inclusion of social support measures alongside all indicators of social capital (Model 6) again improves model fit (AIC) the most, given the strong overall associations between friend/family support and externalizing scores (despite the absence of social support/survey-period moderating associations). The inclusion of weaker-tie dimensions of social capital (which do exhibit moderating associations) (Model 4) represent an improvement in the AIC on the baseline model (Model 1). However, their inclusion also reduces model parsimony (BIC: comparing Model 4 to Models 1–3). Ultimately, while models including extracurricular involvement and neighborhood social capital exhibit slightly poorer fit compared to models only including stronger-tie social capital, the observed moderating relationships of extracurricular involvement and adolescent neighborhood social capital remain substantively important, especially given the absence of stronger-tie moderating associations for externalizing difficulties.

### Fixed Effects Analysis

The second analytic approach aimed to more robustly test the posited stress-buffering role of social capital. Individual fixed effects modelling is applied to two waves of data (2017-18 and November 2020 - in which repeated social capital measures are available), restricted to adolescents who were present in both waves (Table 5). Models contain all covariates although not shown (see Supplementary-Appendix: S.5 for full results).

**Table 5:** Fixed Effects Regression Models Examining Trends in SDQ Internalizing and Externalizing Scores across Levels of Adolescent Social Capital (Longitudinal Panel Approach)

	Model 1	Model 2	Model 3	Model 4	Model 5	Model 6
SDQ type	Internal.	Internal.	Internal.	External.	External.	External.
baseline - Survey wave 2017-18	ref.	ref.	ref.	ref.	ref.	ref.
November 2020	1.419***	1.411***	7.930***	0.829***	0.781***	4.259**
	(0.281)	(0.257)	(1.874)	(0.242)	(0.227)	(1.378)
*Social capital measures*						
Eating family meal (F)		-0.805*	-0.726*		-0.249	-0.165
		(0.375)	(0.325)		(0.198)	(0.214)
November 2020 * Family meal (F)			-0.342			-0.023
			(0.325)			(0.242)
Close friends (N)		-0.045+	-0.030		0.004	0.014
		(0.026)	(0.021)		(0.018)	(0.013)
November 2020 * Close friends (N)			-0.122*			-0.042
			(0.051)			(0.034)
Family support		-0.461	0.879		-0.053	0.081
		(0.694)	(0.752)		(0.496)	(0.412)
November 2020 * Family support			-1.474*			-0.412
			(0.616)			(0.441)
Neighborhood safety		-0.339	-0.156		0.253	0.655*
		(0.207)	(0.231)		(0.149)	(0.217)
November 2020 * Neigh. safety			-0.211			-0.758*
			(0.267)			(0.295)
Constant	4.350**	11.01***	5.195*	5.285***	4.841**	3.772**
	(1.353)	(1.873)	(2.268)	(0.738)	(1.530)	(1.440)
*Within* R-Squared	0.146	0.209	0.249	0.041	0.054	0.116
Observations	1214	1214	1214	1214	1214	1214

F = Frequency; N = Number; SDQ = Strengths and Difficulties Questionnaire; Neigh. = Neighbourhood; Internal. = Internalizing scores; External. = Externalizing scores; UK Household Longitudinal Study Mainstage and UK Household Longitudinal Study COVID-19 data; standard errors in parentheses; models contain all covariates (full results in Supplementary-Appendix: S.5)

+ p < .10, * p < .05, ** p < .01, *** p < .001

Models 1–3 examine internalizing scores. Model 1 (Table 5) demonstrates that internalizing scores significantly increased with the onset of the pandemic within the longitudinal sample. Model 2 includes all available social capital indicators, demonstrating how changes in family/friend networks are associated with changes in internalizing symptoms (changes in family support are not significant after including changes in frequency of family meals). To test for the potential stress-buffering role of social capital, Model 3 adds in the survey-period interaction-terms. The findings mirror the pooled cross-sectional findings. Adolescents whose close friend network increased in size and who felt increasingly supported by their family with the onset of the pandemic experienced significantly smaller increases in internalizing scores. However, neighborhood safety did not moderate the onset of the pandemic (after including friend/family connectivity), while the frequency of family meals moderator was also non-significant. The within r-squared values suggest including the social capital moderating associations improves model fit (Model 3 compared to Model 2). Models 4–6 examine externalizing scores. Model 4 demonstrates that externalizing scores significantly increased with the onset of the pandemic within the longitudinal sample. Model 5 includes available social capital indicators, but none are associated with changes in externalizing symptoms. Model 6 tests the moderating role of social capital and again demonstrates similar results to the pooled cross-sectional findings. Adolescents who felt safer in their neighborhood over the pandemic saw a significantly smaller increase in externalizing scores. The within r-squared values suggest including the social capital moderating associations improves model fit (Model 6 compared to Model 5).

### Robustness Tests

Several sensitivity and robustness tests were undertaken. One issue is whether the pre- to peri-pandemic trends in SDQ represent a pandemic-effect or a continuation of increasing SDQ scores evident before the pandemic. This possibility is tested by looking at changes in SDQ scores between wave 7 (2015-16) and wave 9 (2017-18) of the UK Household Longitudinal Study Mainstage data among adolescents. Both externalizing scores (-0.04 points [-0.20, 0.12]) and internalizing scores (+ 0.11 points [-0.07, 0.28]) exhibit no significant change over the period, suggesting observed pre-/peri-pandemic increases are unlikely to be solely a continuation of an upward pre-pandemic trend.

Another issue is whether the observed social capital moderating associations are driven by associations between social capital and other socio-demographic characteristics. To test for this possibility, the analysis replicates Model 6 (in Table 3 – internalizing – and Table 4 – externalizing) but includes interaction-terms between survey-period and each covariate in the model alongside the survey-period/social capital interaction-terms. For internalizing scores, the findings show larger pre-/peri-pandemic increases among adolescents in single-parent households, with a limiting disability and among females. However, all survey-period/social capital moderators remain significant (see Supplementary-Appendix: S.6). For SDQ externalizing, the results also demonstrate that the social capital moderating interactions remain broadly significant (see Supplementary-Appendix: S.7).

Another possibility is that given the number of interactions in the model, there is a risk of bias from multicollinearity. The variance inflation factor (VIF) scores were obtained for the final models (Model 6 in either Table 3 or Table 4) and were broadly within acceptable boundaries. The mean VIF was 4.82. For most coefficients linked to the social capital/survey-period interactions (the social capital coefficient and its attendant survey-period interaction-terms), VIF scores did not exceed 6.54. The exceptions were the moderation coefficients associated with household-level social capital, which were between 16 and 18, and the VIF scores for the survey-period coefficients, which were between 16 and 17. While this may be expected in the latter case, given the number of interaction tests performed, it does raise concerns. However, all models were re-run excluding the household-level social capital/survey-period interaction-terms. After doing so, no VIF score (including for the survey periods) exceeded 6.54. In addition, the substantive findings reported above remain consistent, even without household-level social capital in the models, suggesting multicollinearity does not substantially bias the models. Results consistent with this discussion were returned across all models reported in the analysis.

The models are also susceptible to bias from reverse causality, where adolescents’ SDQ might condition how the pandemic impacted their social capital, and not vice versa. To explore this possibility, longitudinal fixed effects models are implemented predicting each social capital measure and including interaction-terms between survey-period and internalizing/externalizing scores. Internalizing scores do not moderate changes in social capital over the pandemic. The survey-period/externalizing score interaction-term does significantly predict neighborhood safety. However, this is in the opposite direction than would be expected. These results provide some evidence that the current findings are not solely driven by reverse causality (see Supplementary-Appendix: S.8).

Lastly, the number of hypothesis tests performed (survey-period/social capital interaction-terms) increases risks of false positives. To address this, the Benjamini and Hochberg (1995) correction is applied to the two final full models (Model 6, Table 3 or 4). The analysis takes two approaches to examining the false discovery rate (FDR). The first approach treats each survey-period/social capital interaction-term (three interactions per social capital dimension) as a separate hypothesis, resulting in twenty-four hypotheses. For externalizing symptoms, the neighborhood safety and survey-period interaction-terms (for August 2020 and March 2021) remain significant at an FDR of 10%. For internalizing problems, the survey-period and friend support interaction-terms (for November 2020, March 2021) remain significant at an FDR of 10%, while the survey-period and family support interaction-term (for November 2020) also remain significant at an FDR of 10%. Lastly, the survey-period and close friend network interaction-term (for August 2020) remains significant at an FDR of 10%, but the survey-period and family network interaction-terms are no longer significant. It should be noted, however, that these results for friend/family networks are after accounting for the friend/family social support measures in the model, which already mediate part of the proposed buffering effects of friend/family networks. Given the theoretical grounding of the stated moderation hypotheses and the limited statistical power typically associated with interaction terms, an FDR threshold of 10% is considered to be appropriate. However, at an FDR of 5% only the survey-period/family support interaction (November 2020) and survey-period/friend support interaction (November 2020) remain significant.

Rather than treating every interaction-term between each survey-period and social capital as a separate hypothesis, an alternative approach is taken which seeks to align more with the theoretical aim of testing whether each dimension of social capital moderates overall trends in SDQ over the pandemic in general (rather than moderating each survey-period). To do so, the analysis treats the full set of interaction-terms for any one measure of social capital (e.g., all close friend/survey-period terms) as a single hypothesis. A joint-significance Wald test is then used to assess whether the interaction-terms for any one measure of social capital are simultaneously equal to zero, resulting in eight hypotheses. The Benjamini and Hochberg (1995) correction is then applied to the p-values derived from these joint tests. Under this approach, the neighborhood safety moderating association is statistically significant at an FDR of 10% for externalizing scores. For internalizing scores, the friend support, family support and close friend network size remain significant at an FDR of 10%, and friend/family support remain significant at an FDR of 5%. Taken together, both approaches generally support a moderating role of neighborhood safety for externalizing scores, and a moderating role of friend/family support for internalizing scores, with some evidence of a significant, independent moderating role of close friend networks even after accounting for social support.

## Discussion

Large-scale crises, such as the COVID-19 pandemic, can significantly harm adolescent mental health and leave a lasting scar, even after the initial stressors have abated (Montero-Marin et al., 2023). Understanding what factors can protect adolescent mental health during such crises is thus of high importance. Research demonstrates how social capital is a key stress-buffering resource among adults, reducing the impact of large-scale crises on their mental health. However, little research has investigated whether social capital confers similar protection for adolescent mental health during widespread societal disruptions. Using the COVID-19 pandemic as a case study, this study sought to explore the stress-buffering capacity of different dimensions of adolescent social capital during a large-scale crisis for adolescent emotional-behavioral difficulties, leveraging four waves of UK panel data to track pre- to peri-pandemic changes to in adolescent internalizing and externalizing scores (SDQ). The results demonstrate that adolescent peri-pandemic social capital was linked with attenuated increases in emotional-behavioral difficulties over the pandemic, in part, due to its association with higher perceived social support. However, these apparent stress-buffering effects were not uniform: while some forms of social capital were linked to moderating trajectories in internalizing scores, other forms were linked to moderating externalizing trajectories, and some forms offered no protective effects.

Regarding internalizing difficulties, adolescents who reported more close friends and who regularly shared family meals during the pandemic (indicators of strong-tie friend/family social capital) experienced significantly smaller increases in internalizing difficulties compared to their peers with lower friend/family social capital. A significant portion of the moderating role of strong-tie social capital over the pandemic was explained by its association with the moderating role of perceived social support from family and friends. Adolescents who exhibited greater peri-pandemic family/friend social capital also reported greater perceived family/friend social support, which itself significantly attenuated upward trends in internalizing difficulties. However, even after accounting for their social support from families, and especially from close friends, adolescents’ level of family/friend social capital continued to moderate trends in internalizing difficulties, suggesting social capital buffering pathways outside of social support may be present. An apparent moderating role of perceived neighborhood safety (adolescent-reported neighborhood social capital) was accounted for by its association with strong-tie networks, demonstrating the importance of adjusting estimates for multiple types of social capital to identify which may be driving apparent stress-buffering effects.

In contrast to internalizing symptoms, the findings demonstrate limited evidence that strong-tie social capital buffered externalizing difficulties. Instead, perceptions of neighborhood safety (and to a lesser extent extracurricular involvement) were more consistently associated with attenuating increases in externalizing scores over the pandemic. However, these moderating associations could not be explained by adolescents’ levels of friend/family social support. Indeed, perceived social support itself did not moderate pandemic-related trends in externalizing difficulties. Little support was found across either internalizing or externalizing difficulties for the protective role of parents’ social embeddedness in their neighborhoods (parent reported neighborhood social capital) nor, as observed among adults, a protective role of contextual-level community social capital (Laurence, 2025a; Sato et al., 2022). Longitudinal fixed effects modelling provided more robust evidence of the study’s key findings.

Collectively, the findings provide partial support for the study’s expectations that social capital may buffer the impact of large-scale crises on adolescent internalizing/externalizing difficulties; however, they also demonstrate important specificity regarding its posited protective role. The most consistent supportive evidence relates to the moderating role of strong-tie (close friend/family) social capital and adolescent internalizing scores. These findings align with the broader literature on drivers of internalizing difficulties, which highlights its sensitivity to adolescents’ interpersonal environments, especially whether someone feels connected and supported emotionally (Achenbach et al., 2016). Stronger family and close friend connectivity (social capital) in particular, can provide greater emotional security, feelings of belonging/acceptance, and opportunities for support (Steinberg & Morris, 2001). This may explain why, during the pandemic, it is strong-tie social capital, rather than more diffuse forms (e.g., neighborhood social capital), which cushioned adolescent internalizing symptoms. In line with the stress-buffering model (Cohen & Wills, 1985), these relationships in particular may have afforded more emotional reassurance, appraisal, and coping assistance to buffer pandemic-stressors (particularly social dislocation) on internalizing difficulties (Ellis et al., 2020). Consistent with this interpretation, the findings reveal a significant part of the moderating role of strong tie social capital is accounted for by its association with greater perceived family/friend social support. However, that strong tie social capital continues to moderate internalizing trajectories even after accounting for perceived social support suggests other pathways of buffering linked to strong-tie social capital may also confer protection, such as a stronger sense of belongingness or better developed internalized coping skills.

At the same time, there is little evidence that strong-tie social capital cushioned externalizing difficulties during the pandemic. This appears in line with the broader literature suggesting that while internalizing symptoms are tied more to close relationships, externalizing behaviors may be shaped more by influences outside the home (e.g., neighborhoods) and the types of environments adolescents socialize within (see below) (Loeber & Burke, 2011). In fact, close friend ties can sometimes exacerbate externalizing problems (Loeber & Burke, 2011). This broader point finds additional evidence given perceived friend/family social support did not moderate externalizing trajectories, further suggesting the limits of strong tie social capital for cushioning externalizing behaviors from large-scale crises (even though adolescents reporting more social support did exhibit fewer externalizing problems, i.e., a direct effect of support).

Where evidence of a social capital stress-buffering role is weaker is in relation to adolescents’ externalizing behaviors. Only adolescents’ peri-pandemic perceived neighborhood safety (as an indicator of their own neighborhood social capital) appeared to consistently moderate trajectories in externalizing behaviors over the pandemic. This finding aligns with the broader literature on the importance of neighborhoods in shaping externalizing behaviors (Loeber & Burke, 2011). Feeling safer in one’s neighborhood in particular is believed to reduce externalizing problems via reducing experiences of chronic stress/hypervigilance, providing opportunities for safer exploration and peer interaction, and can reflect the presence of greater local informal social control, which can reinforce prosocial norms, and support more structured socializing (Midouhas et al., 2021). Accordingly, the reduction of more structured socializing and stimulation during the pandemic (e.g., from school closures), coupled with stressors of boredom, disrupted routines and isolation, may have increased externalizing problems (e.g., Levante et al., 2023). However, feeling safer in the neighborhood may have reduced additional environmental stressors (especially given the neighborhood was often the sole space for socializing/interaction), provided greater opportunities to go outside and interact with peers (in more controlled environments), and reduced exposure to difficult home environments, in turn, protecting adolescents from increasing externalizing problems.

Where the findings do not conform to the study’s expectations, and also point to the wider limitations of neighborhood social capital for protection, is the absence of any stress-buffering (especially for externalizing problems) by how socially embedded adolescents’ parents were in the local area or in the average level of social capital in adolescents’ wider residential community (contextual-level social capital). From one perspective, this is surprising, especially given the strong link between externalizing behaviors and neighborhood conditions, where neighborhood parental- and contextual-level social capital are posited to foster greater informal social control, reinforce prosocial norms, and support more structured socializing (Loeber & Burke, 2011; Visser et al., 2021). However, one reason parental/contextual-level neighborhood social capital did not attenuate increases in externalizing behaviors may be that pandemic restrictions reduced adolescents’ time in their neighborhoods, thereby limiting opportunities for informal social control, the reinforcement of prosocial norms, and positive peer modelling to operate as protective mechanisms. Another possibility is that restrictions also curtailed neighbors’ wider community participation, inhibiting usual processes of social engagement and monitoring which reduces adolescents externalizing behaviors. Under such conditions of reduced community engagement, the protective role of neighborhood social capital against externalizing difficulties may have depended much more on adolescents’ perceptions of their neighborhood (their own neighborhood social capital), which could have become more salient as the role of actual social structures weakened. Interestingly, despite expectations, the findings demonstrate mostly limited protection conferred by adolescent extra-curricular involvement. This may stem from the fact that this measure captured ‘usual’ involvement in activities and, during the pandemic, disruptions to these routines may have weakened their ability to protect adolescents from the impact of stressors, e.g., reduced time in structured, supervised, prosocial spaces or separation from prosocial peer groups.

Taken together, the findings provide conditional support for the role of social capital as a stress-buffer for adolescent internalizing/externalizing difficulties during large-scale crises. However, different forms of social capital appear to confer greater/lesser protection against different clusters of internalizing/externalizing problems. These findings thus suggest adolescent stress-buffering processes during crises may operate somewhat differently than for adults, especially given the lack of evidence for the well-documented protective role of community (Laurence & Kim, 2021) and regional (Sato et al., 2022) social capital for adult mental health during crises. Instead, strong-tie social capital (especially friends/family ties) appears particularly effective for protecting adolescents, and primarily from internalizing problems.

Despite these insights, this study has several limitations. Measures of adolescents’ peri-pandemic social capital were only available in November 2020. Relative temporal stability in adolescents’ social capital in August 2020 and March 2021 is thus assumed, which may not fully capture fluctuations in social experiences during a period of rapidly shifting restrictions. Relatedly, the study could not assess SDQ/social capital immediately prior to the onset of the pandemic (with baseline data only available in 2017-18), raising the possibility that SDQ had already started worsening after 2018. The fixed effects analyses also remain susceptible to bias from time variant unobserved heterogeneity. In addition, there is potential bias from endogeneity between social capital and SDQ (whether the former cushions the latter, or vice versa). As mentioned above, the sample restrictions to adolescents who participated in the November 2020 wave of the UK Household Longitudinal Study COVID-19 study may also bias estimates due to non-response and attrition, especially given non-responders tended to come from more disadvantaged environments. Although weighting strategies were undertaken, the possibility remains the current findings are biased towards less disadvantaged adolescents. Key questions also remain regarding the mechanisms explaining any stress-buffering role of social capital. While demonstrating that the apparent stress-buffering role of strong-tie social capital is explained by its association with perceived support, issues of endogeneity remain given both are self-reported at the same time-point. Furthermore, instead of being viewed as a mediator, perceived support may itself be conceived as an indicator of social capital (tie relational quality), leaving the question open as to what pathways explain the cushioning findings for internalizing difficulties. In addition, the data did not allow us to examine what pathways might account for the apparent stress-buffering role of neighborhood safety for externalizing problems. Finally, the data did not contain measures of school-based social capital, which is a potentially key source of adolescent social capital (McPherson et al., 2014).

## Conclusion

As concern grows that the risk of major societal crises is increasing, and evidence that repeat exposure to such crises can have compounding impacts on adolescent mental health, it is critical to identify what factors can protect adolescents from harm during major disruptions. Examining this question through the context of the COVID-19 pandemic, this study explored the stress-buffering capacity of adolescent social capital during large-scale crises for emotional-behavioral symptomology by tracking trends in internalizing/externalizing difficulties over four waves of UK panel data and exploring if, how and why these trends differed according to adolescents’ peri-pandemic social capital. The findings demonstrate promising evidence that social capital can attenuate rises in emotional-behavioral problems following societal disruptions, in part, through access to greater perceived social support. However, different dimensions of social capital exhibit different capacities for cushioning emotional or behavioral difficulties. While greater strong-tie (family/friend) social capital attenuated increases in internalizing difficulties over the pandemic, it was largely adolescents’ neighborhood social capital which attenuated rises in externalizing difficulties. Meanwhile, extracurricular engagement, social capital among parents, and social capital between residents in adolescents’ wider community, conferred more limited protection, at least during the COVID-19 pandemic. These findings demonstrate how young people’s social capital can be an important psychosocial buffering factor in adolescence during large-scale crises, especially for their emotional symptomology. However, the findings also demonstrate how the protective capacity of psychosocial buffering resources appears to be shaped by the differing etiologies and contextual sensitivities of emotional and behavioral difficulties. These insights have implications for youth crisis-preparedness policy, which needs to reflect this conditional relationship between different forms of social capital and clusters of emotional-behavioral outcomes during crises.

## Supplementary Information

Below is the link to the electronic supplementary material.

Supplementary Material 1

## Declarations

### Data Sharing Declaration

Data in the study are from Understanding Society: The UK Household Longitudinal Study, which is led by the Institute for Social and Economic Research at the University of Essex and funded by the Economic and Social Research Council [Grant Number: ES/M008592/1]. The datasets analyzed during the current study are publicly available in the UK Data Service repository: ‘Understanding Society: Waves 1-14, 2009-2023 and Harmonised BHPS: Waves 1-18, 1991-2009’ (10.5255/UKDA-SN-6614-20) and ‘Understanding Society: COVID-19 Study, 2020-2021’ (10.5255/UKDA-SN-8644-11). Restrictions apply to the data containing merged geographic identifiers which can only be accessed under more restrictive conditions: ‘Understanding Society: Waves 1-14, 2009-2023: Special Licence Access, Census 2021 Lower Layer Super Output Areas’ (10.5255/UKDA-SN-9169-3) and ‘Understanding Society: COVID-19 Study, 2020-2021: Special Licence Access, Census 2011 Lower Layer Super Output Areas’ (10.5255/UKDA-SN-8663-7).
