# Supplementary material for: Adolescent Social Capital as a Source of Resilience against Emotional and Behavioural Difficulties in Times of Crisis: Longitudinal Evidence from the COVID-19 Pandemic
Source: J Youth Adolesc. 2025 Dec 4;55(4):998–1020. doi: 10.1007/s10964-025-02295-5 (PMC13076373; doi:10.1007/s10964-025-02295-5)
Supplement: Supplementary file 1 — Supplementary Material 1 [file 10964_2025_2295_MOESM1_ESM.docx]

**Supplementary Analysis**

**Adolescent Social Capital as a Source of Resilience Against Emotional and Behavioural Difficulties in Times of Crisis: Longitudinal Evidence From the COVID-19 Pandemic**

**James Laurence & Bill Calvey**

**Table S.1 – Descriptive statistics of sample used in the pooled cross-sectional analysis (uncentered values reported)**

|  | Mean | Minimum | Maximum | Standard deviation |
| --- | --- | --- | --- | --- |
| SDQ Internalising | 5.062 | 0 | 19 | 3.532 |
| SDQ Externalising | 5.694 | 0 | 18 | 3.582 |
| Economic disadvantage (index) | 0.142 | 1.788 | 2.323 | 0.886 |
| Social disadvantage (index) | 0.075 | 3.683 | 1.622 | 0.899 |
| Eating family meal (F) | 3.433 | 1 | 4 | 0.84 |
| Close friends (N) | 6.088 | 0 | 70 | 5.054 |
| Family support | 2.772 | 1 | 3 | 0.454 |
| Friend support | 2.713 | 1 | 3 | 0.502 |
| Neighbourhood safety | 2.67 | 1 | 4 | 0.844 |
| Extra-curricular (N) | 0.556 | 0 | 6 | 0.847 |
| Household social capital | 3.52 | 1 | 5 | 0.869 |
| Local Authority Relationships Index | 3.719 | 1.886 | 5.942 | 0.832 |
| Local Authority COVID-19 case rate | 69.83 | 0 | 599.7 | 87.03 |
|  |  |  |  |  |
|  | % |  |  |  |
| Household education |  |  |  |  |
| No degrees in HH | 53.9% |  |  |  |
| Degree in HH | 46.1% |  |  |  |
| Household n of adults in employment |  |  |  |  |
| 0 | 8.6% |  |  |  |
| 1 | 49.2% |  |  |  |
| 2+ | 42.1% |  |  |  |
| Household financial situation |  |  |  |  |
| Not coping well financially | 22.7% |  |  |  |
| Coping well financially | 77.3% |  |  |  |
| Living with partner in HH |  |  |  |  |
| Not living with partner | 17.1% |  |  |  |
| Living with partner | 82.9% |  |  |  |
| Youth disability status |  |  |  |  |
| No | 87.6% |  |  |  |
| Limited a little | 10.5% |  |  |  |
| Limited a lot | 1.9% |  |  |  |
| Youth ethnic group |  |  |  |  |
| White | 78.5% |  |  |  |
| Mixed | 5.7% |  |  |  |
| Asian | 10.5% |  |  |  |
| Other | 2.6% |  |  |  |
| Black | 2.7% |  |  |  |
| Youth gender |  |  |  |  |
| Male | 46.8% |  |  |  |
| Female | 53.2% |  |  |  |
| Youth age |  |  |  |  |
| 10-11 | 37.6% |  |  |  |
| 12-13 | 34.7% |  |  |  |
| 14-15 | 27.7% |  |  |  |
| N (observations) | 3,176 |  |  |  |

**Table S.2 – Descriptive statistics of sample used in the longitudinal analysis (uncentered values reported)**

|  | Mean | Minimum | Maximum | Standard deviation |
| --- | --- | --- | --- | --- |
| SDQ Internalising | 5.062 | 0 | 18 | 3.583 |
| SDQ Externalising | 5.715 | 0 | 18 | 3.568 |
| Economic disadvantage (index) | 0.107 | 1.788 | 2.323 | 0.902 |
| Social disadvantage (index) | 0.084 | 3.683 | 1.622 | 0.939 |
| Eating family meal (F) | 3.38 | 1 | 4 | 0.869 |
| Close friends (N) | 5.98 | 0 | 42 | 4.85 |
| Family support | 2.73 | 1 | 3 | 0.476 |
| Friend support | 2.713 | 1 | 3 | 0.506 |
| Neighbourhood safety | 2.713 | 1 | 4 | 0.843 |
| Extra-curricular (N) | 0.499 | 0 | 5 | 0.802 |
| Household social capital | 2.993 | 0 | 5 | 1.497 |
| Local Authority Relationships Index | 3.725 | 1.886 | 5.942 | 0.816 |
| Local Authority COVID-19 case rate | 45.76 | 0 | 560.2 | 76.49 |
|  |  |  |  |  |
|  | % |  |  |  |
| Household education |  |  |  |  |
| No degrees in HH | 59.9% |  |  |  |
| Degree in HH | 40.1% |  |  |  |
| Household n of adults in employment |  |  |  |  |
| 0 | 7.4% |  |  |  |
| 1 | 40.4% |  |  |  |
| 2+ | 52.1% |  |  |  |
| Household financial situation |  |  |  |  |
| Not coping well financially | 24.7% |  |  |  |
| Coping well financially | 75.3% |  |  |  |
| Living with partner in HH |  |  |  |  |
| Not living with partner | 20.7% |  |  |  |
| Living with partner | 79.3% |  |  |  |
| Youth disability status |  |  |  |  |
| No | 86.6% |  |  |  |
| Limited a little | 11.3% |  |  |  |
| Limited a lot | 2.2% |  |  |  |
| Youth ethnic group |  |  |  |  |
| White | 73.2% |  |  |  |
| Mixed | 5.7% |  |  |  |
| Asian | 15.3% |  |  |  |
| Other | 2.1% |  |  |  |
| Black | 3.6% |  |  |  |
| Youth gender |  |  |  |  |
| Male | 47.2% |  |  |  |
| Female | 52.8% |  |  |  |
| Youth age |  |  |  |  |
| 10-11 | 30.1% |  |  |  |
| 12-13 | 39.3% |  |  |  |
| 14-15 | 30.6% |  |  |  |
| N (observations) | 1,214 |  |  |  |

**Table S.3 - Full model results for multilevel mixed regressions examining trends in SDQ internalising scores across levels of adolescent social capital (pooled cross-sectional approach)**

|  | Model 1 | Model 2 | Model 3 | Model 4 | Model 5 | Model 6 |
| --- | --- | --- | --- | --- | --- | --- |
| SDQ type | Internal. | Internal. | Internal. | Internal. | Internal. | Internal. |
|  |  |  |  |  |  |  |
| baseline - No degree in HH | ref. | ref. | ref. | ref. | ref. | ref. |
| Degree in HH | -0.410+ | -0.401+ | -0.408+ | -0.415* | -0.416* | -0.296 |
|  | (0.217) | (0.210) | (0.212) | (0.208) | (0.205) | (0.186) |
| baseline - None employed in HH | ref. | ref. | ref. | ref. | ref. | ref. |
| 1 | 0.290 | 0.352 | 0.293 | 0.322 | 0.326 | 0.404 |
|  | (0.260) | (0.261) | (0.263) | (0.267) | (0.268) | (0.272) |
| 2+ | 0.185 | 0.222 | 0.225 | 0.168 | 0.213 | 0.301 |
|  | (0.294) | (0.296) | (0.294) | (0.295) | (0.295) | (0.298) |
| baseline - HH not coping financially | ref. | ref. | ref. | ref. | ref. | ref. |
| HH coping well financially | -0.313 | -0.291 | -0.269 | -0.286 | -0.251 | -0.166 |
|  | (0.225) | (0.223) | (0.222) | (0.226) | (0.226) | (0.213) |
| baseline - single parent HH | ref. | ref. | ref. | ref. | ref. | ref. |
| Partner in HH | -0.033 | -0.011 | -0.013 | -0.023 | -0.014 | 0.013 |
|  | (0.177) | (0.178) | (0.172) | (0.174) | (0.170) | (0.176) |
| baseline - Not limited by disability | ref. | ref. | ref. | ref. | ref. | ref. |
| Limited a little | 2.142*** | 1.949*** | 2.140*** | 1.917*** | 1.943*** | 1.492*** |
|  | (0.330) | (0.309) | (0.318) | (0.316) | (0.310) | (0.310) |
| Limited a lot | 3.472*** | 2.734*** | 3.061*** | 3.020*** | 2.683*** | 2.224*** |
|  | (0.759) | (0.593) | (0.695) | (0.665) | (0.600) | (0.492) |
| baseline - White | ref. | ref. | ref. | ref. | ref. | ref. |
| Mixed | -0.564 | -0.521 | -0.378 | -0.670+ | -0.491 | -0.604* |
|  | (0.357) | (0.332) | (0.332) | (0.344) | (0.325) | (0.282) |
| Asian | -0.758* | -0.974** | -0.814** | -0.919** | -0.954** | -0.968** |
|  | (0.315) | (0.311) | (0.307) | (0.319) | (0.310) | (0.295) |
| Other | 0.260 | -0.044 | 0.303 | -0.109 | -0.073 | 0.179 |
|  | (0.604) | (0.622) | (0.652) | (0.569) | (0.616) | (0.567) |
| Black | -0.480 | -1.153* | -0.933 | -0.688 | -1.111+ | -0.855 |
|  | (0.647) | (0.588) | (0.647) | (0.588) | (0.602) | (0.541) |
| baseline - Male | ref. | ref. | ref. | ref. | ref. | ref. |
| Female | 1.086*** | 0.692*** | 1.015*** | 0.742*** | 0.710*** | 0.791*** |
|  | (0.170) | (0.161) | (0.161) | (0.169) | (0.161) | (0.155) |
| baseline - Aged 10-11 | ref. | ref. | ref. | ref. | ref. | ref. |
| 12-13 | -0.025 | 0.041 | 0.005 | 0.074 | 0.090 | 0.075 |
|  | (0.184) | (0.182) | (0.177) | (0.184) | (0.179) | (0.173) |
| 14-15 | 0.547* | 0.667** | 0.468* | 0.802*** | 0.692** | 0.566** |
|  | (0.235) | (0.225) | (0.217) | (0.229) | (0.216) | (0.200) |
| LA Economic disadvantage | -0.045 | -0.101 | 0.012 | -0.137 | -0.082 | -0.097 |
|  | (0.138) | (0.171) | (0.138) | (0.164) | (0.165) | (0.160) |
| LA Social disadvantage | 0.387** | 0.293+ | 0.322* | 0.366* | 0.305+ | 0.270+ |
|  | (0.148) | (0.156) | (0.143) | (0.157) | (0.156) | (0.150) |
| LA Social disadvantage * LA Social disadvantage | 0.174** | 0.162** | 0.160* | 0.180** | 0.165** | 0.146* |
|  | (0.063) | (0.062) | (0.065) | (0.060) | (0.062) | (0.061) |
| LA COVID-19 case rate | -0.000 | -0.000 | -0.000 | -0.000 | -0.000 | -0.000 |
|  | (0.000) | (0.000) | (0.000) | (0.000) | (0.000) | (0.000) |
| baseline - Survey wave 2017-18 | ref. | ref. | ref. | ref. | ref. | ref. |
| August 2020 | 0.491* | 0.405+ | 2.341*** | 1.554 | 2.722* | 5.717*** |
|  | (0.223) | (0.221) | (0.677) | (1.238) | (1.334) | (1.427) |
| November 2020 | 0.570** | 0.482* | 3.018*** | 2.101 | 3.648** | 8.708*** |
|  | (0.218) | (0.220) | (0.721) | (1.389) | (1.361) | (1.587) |
| March 2021 | 0.575** | 0.492* | 3.409*** | 1.945 | 4.095** | 7.117*** |
|  | (0.218) | (0.216) | (0.721) | (1.388) | (1.345) | (1.522) |
| *Social capital measures* |  |  |  |  |  |  |
| Eating family meal (F) |  | -0.392*** | -0.058 |  | -0.065 | 0.031 |
|  |  | (0.111) | (0.179) |  | (0.177) | (0.177) |
| August 2020 * Eating family meal (F) |  |  | -0.311 |  | -0.301 | -0.136 |
|  |  |  | (0.192) |  | (0.194) | (0.204) |
| November 2020 * Eating family meal (F) |  |  | -0.493* |  | -0.468* | -0.206 |
|  |  |  | (0.203) |  | (0.207) | (0.201) |
| March 2021 * Eating family meal (F) |  |  | -0.594** |  | -0.570** | -0.461* |
|  |  |  | (0.199) |  | (0.199) | (0.203) |
| Close friends (N) |  | -0.105*** | -0.031 |  | -0.026 | -0.010 |
|  |  | (0.019) | (0.040) |  | (0.039) | (0.040) |
| August 2020 * Close friends (N) |  |  | -0.123** |  | -0.113** | -0.094* |
|  |  |  | (0.039) |  | (0.038) | (0.039) |
| November 2020 * Close friends (N) |  |  | -0.117** |  | -0.104* | -0.082+ |
|  |  |  | (0.044) |  | (0.043) | (0.042) |
| March 2021 * Close friends (N) |  |  | -0.122** |  | -0.110** | -0.092* |
|  |  |  | (0.044) |  | (0.042) | (0.044) |
| Neighbourhood safety |  | -0.742*** |  | -0.508* | -0.532** | -0.459* |
|  |  | (0.119) |  | (0.208) | (0.200) | (0.205) |
| August 2020 * Neighbourhood safety |  |  |  | -0.377+ | -0.231 | -0.150 |
|  |  |  |  | (0.215) | (0.209) | (0.215) |
| November 2020 * Neighbourhood safety |  |  |  | -0.470* | -0.305 | -0.166 |
|  |  |  |  | (0.229) | (0.221) | (0.215) |
| March 2021 * Neighbourhood safety |  |  |  | -0.462* | -0.265 | -0.190 |
|  |  |  |  | (0.236) | (0.237) | (0.247) |
| HH social capital |  | -0.041 |  | -0.033 | -0.034 | -0.052 |
|  |  | (0.086) |  | (0.096) | (0.095) | (0.092) |
| August 2020 * HH Social capital |  |  |  | -0.054 | -0.036 | 0.037 |
|  |  |  |  | (0.221) | (0.217) | (0.214) |
| November 2020 * HH Social capital |  |  |  | -0.007 | 0.023 | 0.120 |
|  |  |  |  | (0.247) | (0.250) | (0.224) |
| March 2021 * HH Social capital |  |  |  | -0.012 | -0.029 | -0.000 |
|  |  |  |  | (0.228) | (0.232) | (0.227) |
| LA Relationships Index |  | -0.093 |  | -0.042 | -0.074 | -0.164 |
|  |  | (0.210) |  | (0.272) | (0.271) | (0.267) |
| August 2020 * LA Relationships Index |  |  |  | 0.006 | 0.035 | 0.010 |
|  |  |  |  | (0.224) | (0.222) | (0.224) |
| November 2020 * LA Relationships Index |  |  |  | -0.087 | -0.041 | -0.101 |
|  |  |  |  | (0.220) | (0.209) | (0.207) |
| March 2021 * LA Relationships Index |  |  |  | -0.056 | -0.025 | -0.022 |
|  |  |  |  | (0.200) | (0.189) | (0.190) |
| Extra-curricular (N) |  | 0.194 |  | 0.279 | 0.270 | 0.274 |
|  |  | (0.107) |  | (0.192) | (0.185) | (0.183) |
| August 2020 * Extra-curricular (N) |  |  |  | -0.067 | -0.001 | -0.008 |
|  |  |  |  | (0.206) | (0.202) | (0.203) |
| November 2020 * Extra-curricular (N) |  |  |  | -0.178 | -0.116 | -0.112 |
|  |  |  |  | (0.215) | (0.209) | (0.207) |
| March 2021 *Extra-curricular (N) |  |  |  | -0.218 | -0.146 | -0.153 |
|  |  |  |  | (0.222) | (0.215) | (0.221) |
| Family support |  |  |  |  |  | -0.747* |
|  |  |  |  |  |  | (0.360) |
| August 2020 * Family support |  |  |  |  |  | -0.789+ |
|  |  |  |  |  |  | (0.403) |
| November 2020 * Family support |  |  |  |  |  | -1.414*** |
|  |  |  |  |  |  | (0.396) |
| March 2021 * Family support |  |  |  |  |  | -0.426 |
|  |  |  |  |  |  | (0.483) |
| Friend support |  |  |  |  |  | -0.492 |
|  |  |  |  |  |  | (0.330) |
| August 2020 * Friend support |  |  |  |  |  | -0.670* |
|  |  |  |  |  |  | (0.338) |
| November 2020 * Friend support |  |  |  |  |  | -0.969** |
|  |  |  |  |  |  | (0.345) |
| March 2021 * Friend support |  |  |  |  |  | -0.954* |
|  |  |  |  |  |  | (0.376) |
|  |  |  |  |  |  |  |
| Constant | 3.858*** | 8.409*** | 4.227*** | 5.542*** | 6.083*** | 9.060*** |
|  | (0.352) | (0.972) | (0.719) | (1.208) | (1.302) | (1.488) |
| AIC | 14972.75 | 14874.67 | 14873.32 | 14930.85 | 14850.31 | 14652.26 |
| BIC | 15160.71 | 15099.01 | 15109.79 | 15215.83 | 15183.79 | 15034.25 |
| Observations | 3176 | 3176 | 3176 | 3176 | 3176 | 3176 |

*Notes*: F=Frequency; N=Number; HH=Household; LA=Local Authority; SDQ=Strengths and Difficulties Questionnaire; Internal.=Internalizing scores; UK Household Longitudinal Study Mainstage and UK Household Longitudinal Study COVID-19 data; standard errors in parentheses.

+ p<.10, * p<.05, ** p<.01, *** p<.001

**Table S.4 - Full model results for multilevel mixed regressions examining trends in SDQ externalising scores across levels of adolescent social capital (pooled cross-sectional approach)**

|  | Model 1 | Model 2 | Model 3 | Model 4 | Model 5 | Model 6 |
| --- | --- | --- | --- | --- | --- | --- |
| SDQ type | External. | External. | External. | External. | External. | External. |
|  |  |  |  |  |  |  |
| baseline - No degree in HH | ref. | ref. | ref. | ref. | ref. | ref. |
| Degree in HH | -0.572** | -0.521* | -0.533* | -0.541* | -0.500* | -0.430* |
|  | (0.221) | (0.216) | (0.213) | (0.212) | (0.205) | (0.197) |
| baseline - None employed in HH | ref. | ref. | ref. | ref. | ref. | ref. |
| 1 | 0.482+ | 0.519* | 0.482+ | 0.462+ | 0.461+ | 0.509* |
|  | (0.250) | (0.251) | (0.252) | (0.254) | (0.257) | (0.250) |
| 2+ | 0.347 | 0.385 | 0.368 | 0.301 | 0.320 | 0.370 |
|  | (0.274) | (0.271) | (0.271) | (0.276) | (0.275) | (0.269) |
| baseline - HH not coping financially | ref. | ref. | ref. | ref. | ref. | ref. |
| HH coping well financially | -0.244 | -0.209 | -0.196 | -0.225 | -0.183 | -0.129 |
|  | (0.194) | (0.193) | (0.193) | (0.191) | (0.191) | (0.189) |
| baseline - single parent HH | ref. | ref. | ref. | ref. | ref. | ref. |
| Partner in HH | 0.103 | 0.126 | 0.114 | 0.124 | 0.130 | 0.157 |
|  | (0.180) | (0.180) | (0.183) | (0.180) | (0.182) | (0.189) |
| baseline - Not limited by disability | ref. | ref. | ref. | ref. | ref. | ref. |
| Limited a little | 1.563*** | 1.460*** | 1.543*** | 1.444*** | 1.433*** | 1.066** |
|  | (0.333) | (0.338) | (0.338) | (0.336) | (0.340) | (0.341) |
| Limited a lot | 3.305*** | 2.826*** | 2.992*** | 3.087*** | 2.800*** | 2.377*** |
|  | (0.508) | (0.540) | (0.563) | (0.487) | (0.548) | (0.603) |
| baseline - White | ref. | ref. | ref. | ref. | ref. | ref. |
| Mixed | -0.036 | -0.022 | 0.057 | -0.075 | 0.003 | -0.058 |
|  | (0.349) | (0.347) | (0.335) | (0.361) | (0.347) | (0.321) |
| Asian | -0.630* | -0.671* | -0.605* | -0.712* | -0.675* | -0.673* |
|  | (0.302) | (0.315) | (0.297) | (0.324) | (0.321) | (0.310) |
| Other | -0.018 | -0.222 | -0.052 | -0.135 | -0.171 | 0.006 |
|  | (0.621) | (0.651) | (0.622) | (0.648) | (0.642) | (0.638) |
| Black | -0.432 | -0.959+ | -0.876 | -0.470 | -0.885 | -0.646 |
|  | (0.537) | (0.582) | (0.596) | (0.522) | (0.588) | (0.604) |
| baseline - Male | ref. | ref. | ref. | ref. | ref. | ref. |
| Female | -0.521** | -0.697*** | -0.555** | -0.642*** | -0.663*** | -0.593** |
|  | (0.188) | (0.192) | (0.187) | (0.193) | (0.190) | (0.186) |
| baseline - Aged 10-11 | ref. | ref. | ref. | ref. | ref. | ref. |
| 12-13 | 0.044 | 0.051 | 0.032 | 0.083 | 0.064 | 0.021 |
|  | (0.191) | (0.190) | (0.190) | (0.186) | (0.185) | (0.186) |
| 14-15 | 0.214 | 0.224 | 0.149 | 0.371 | 0.307 | 0.209 |
|  | (0.241) | (0.240) | (0.237) | (0.238) | (0.237) | (0.236) |
| LA Economic disadvantage | -0.183 | -0.239 | -0.149 | -0.232 | -0.223 | -0.236 |
|  | (0.152) | (0.180) | (0.148) | (0.183) | (0.182) | (0.181) |
| LA Social disadvantage | 0.365* | 0.270 | 0.325* | 0.344+ | 0.294 | 0.261 |
|  | (0.162) | (0.185) | (0.158) | (0.190) | (0.186) | (0.184) |
| LA Social disadvantage * LA Social disadvantage | 0.125+ | 0.121 | 0.119 | 0.132+ | 0.126+ | 0.111 |
|  | (0.074) | (0.074) | (0.073) | (0.074) | (0.073) | (0.071) |
| LA COVID-19 case rate | -0.002+ | -0.002+ | -0.002+ | -0.002 | -0.002+ | -0.001 |
|  | (0.001) | (0.001) | (0.001) | (0.002) | (0.002) | (0.002) |
| baseline - Survey wave 2017-18 | ref. | ref. | ref. | ref. | ref. | ref. |
| August 2020 | 0.254 | 0.248 | 0.645 | 1.836+ | 1.880 | 0.963 |
|  | (0.206) | (0.205) | (0.837) | (1.069) | (1.285) | (1.505) |
| November 2020 | 0.857** | 0.862** | 1.630* | 2.191+ | 2.633+ | 4.333* |
|  | (0.289) | (0.287) | (0.694) | (1.299) | (1.388) | (1.683) |
| March 2021 | 0.453* | 0.458* | 2.075** | 2.161+ | 3.527** | 4.909** |
|  | (0.219) | (0.217) | (0.764) | (1.217) | (1.363) | (1.641) |
| *Social capital measures* |  |  |  |  |  |  |
| Eating family meal (F) |  | -0.549*** | -0.411* |  | -0.433** | -0.258 |
|  |  | (0.112) | (0.169) |  | (0.167) | (0.174) |
| August 2020 * Eating family meal (F) |  |  | -0.035 |  | 0.002 | -0.079 |
|  |  |  | (0.225) |  | (0.222) | (0.227) |
| November 2020 * Eating family meal (F) |  |  | -0.179 |  | -0.137 | -0.031 |
|  |  |  | (0.193) |  | (0.191) | (0.191) |
| March 2021 * Eating family meal (F) |  |  | -0.410+ |  | -0.367+ | -0.297 |
|  |  |  | (0.211) |  | (0.209) | (0.209) |
| Close friends (N) |  | -0.010 | 0.002 |  | -0.002 | 0.019 |
|  |  | (0.017) | (0.032) |  | (0.032) | (0.032) |
| August 2020 * Close friends (N) |  |  | -0.040 |  | -0.021 | -0.025 |
|  |  |  | (0.035) |  | (0.035) | (0.036) |
| November 2020 * Close friends (N) |  |  | -0.019 |  | -0.004 | 0.001 |
|  |  |  | (0.042) |  | (0.040) | (0.040) |
| March 2021 * Close friends (N) |  |  | -0.027 |  | -0.009 | -0.004 |
|  |  |  | (0.040) |  | (0.040) | (0.039) |
| Neighbourhood safety |  | -0.355** |  | 0.011 | 0.009 | 0.120 |
|  |  | (0.114) |  | (0.167) | (0.167) | (0.164) |
| August 2020 * Neighbourhood safety |  |  |  | -0.487** | -0.455* | -0.523** |
|  |  |  |  | (0.186) | (0.187) | (0.188) |
| November 2020 * Neighbourhood safety |  |  |  | -0.421* | -0.397* | -0.337+ |
|  |  |  |  | (0.193) | (0.185) | (0.183) |
| March 2021 * Neighbourhood safety |  |  |  | -0.599** | -0.556** | -0.502** |
|  |  |  |  | (0.190) | (0.190) | (0.195) |
| HH social capital |  | -0.024 |  | 0.049 | 0.061 | 0.053 |
|  |  | (0.100) |  | (0.112) | (0.114) | (0.112) |
| August 2020 * HH Social capital |  |  |  | -0.351+ | -0.350+ | -0.357+ |
|  |  |  |  | (0.190) | (0.190) | (0.194) |
| November 2020 * HH Social capital |  |  |  | -0.188 | -0.183 | -0.112 |
|  |  |  |  | (0.227) | (0.227) | (0.227) |
| March 2021 * HH Social capital |  |  |  | -0.194 | -0.223 | -0.200 |
|  |  |  |  | (0.213) | (0.214) | (0.215) |
| LA Relationships Index |  | -0.137 |  | -0.207 | -0.246 | -0.341 |
|  |  | (0.241) |  | (0.284) | (0.281) | (0.277) |
| August 2020 * LA Relationships Index |  |  |  | 0.261 | 0.265 | 0.335+ |
|  |  |  |  | (0.188) | (0.186) | (0.187) |
| November 2020 * LA Relationships Index |  |  |  | 0.146 | 0.145 | 0.172 |
|  |  |  |  | (0.169) | (0.166) | (0.161) |
| March 2021 * LA Relationships Index |  |  |  | 0.158 | 0.149 | 0.166 |
|  |  |  |  | (0.179) | (0.177) | (0.175) |
| Extra-curricular (N) |  | -0.007 |  | 0.192 | 0.189 | 0.185 |
|  |  | (0.113) |  | (0.158) | (0.157) | (0.159) |
| August 2020 * Extra-curricular (N) |  |  |  | -0.196 | -0.177 | -0.153 |
|  |  |  |  | (0.183) | (0.181) | (0.174) |
| November 2020 * Extra-curricular (N) |  |  |  | -0.358* | -0.337* | -0.337* |
|  |  |  |  | (0.166) | (0.165) | (0.165) |
| March 2021 *Extra-curricular (N) |  |  |  | -0.201 | -0.167 | -0.195 |
|  |  |  |  | (0.203) | (0.194) | (0.194) |
| Family support |  |  |  |  |  | -1.112** |
|  |  |  |  |  |  | (0.377) |
| August 2020 * Family support |  |  |  |  |  | 0.613 |
|  |  |  |  |  |  | (0.498) |
| November 2020 * Family support |  |  |  |  |  | -0.714 |
|  |  |  |  |  |  | (0.480) |
| March 2021 * Family support |  |  |  |  |  | -0.541 |
|  |  |  |  |  |  | (0.520) |
| Friend support |  |  |  |  |  | -0.608* |
|  |  |  |  |  |  | (0.302) |
| August 2020 * Friend support |  |  |  |  |  | -0.185 |
|  |  |  |  |  |  | (0.394) |
| November 2020 * Friend support |  |  |  |  |  | -0.288 |
|  |  |  |  |  |  | (0.365) |
| March 2021 * Friend support |  |  |  |  |  | -0.161 |
|  |  |  |  |  |  | (0.391) |
|  |  |  |  |  |  |  |
| Constant | 5.429*** | 8.923*** | 6.781*** | 5.999*** | 7.558*** | 11.508*** |
|  | (0.371) | (1.129) | (0.696) | (1.291) | (1.380) | (1.611) |
| AIC | 14964.08 | 14940.52 | 14940.95 | 14954.04 | 14935.43 | 14834.01 |
| BIC | 15152.04 | 15164.87 | 15177.42 | 15239.02 | 15268.91 | 15216.01 |
| Observations | 3176 | 3176 | 3176 | 3176 | 3176 | 3176 |

*Notes*: F=Frequency; N=Number; HH=Household; LA=Local Authority; SDQ=Strengths and Difficulties Questionnaire; External.=Externalizing scores; UK Household Longitudinal Study Mainstage and UK Household Longitudinal Study COVID-19 data; standard errors in parentheses.

+ p<.10, * p<.05, ** p<.01, *** p<.001

**Table S.5 – Full model results for fixed effects regression models examining trends in SDQ internalising and externalising scores across levels of adolescent social capital (longitudinal panel approach)**

|  | Model 1 | Model 2 | Model 3 | Model 4 | Model 5 | Model 6 |
| --- | --- | --- | --- | --- | --- | --- |
| SDQ type | Internalize | Internalize | Internalize | Externalise | Externalise | Externalise |
|  |  |  |  |  |  |  |
| baseline - No degree in HH | ref. | ref. | ref. | ref. | ref. | ref. |
| Degree in HH | 3.166 | 0.311 | 1.838+ | 0.801 | 0.127 | 0.376 |
|  | (2.109) | (0.574) | (0.964) | (0.542) | (0.578) | (0.450) |
| baseline - None employed in HH | ref. |  | ref. | ref. | ref. | ref. |
| 1 | 0.082 | 0.062 | -0.080 | 0.754 | 0.467 | 0.714 |
|  | (0.630) | (0.813) | (0.680) | (0.704) | (0.665) | (0.659) |
| 2+ | 0.587 | 0.421 | 0.716 | 0.830 | 0.668 | 0.984 |
|  | (0.869) | (0.848) | (0.842) | (0.696) | (0.690) | (0.644) |
| baseline - HH not coping financially | ref. |  | ref. | ref. | ref. | ref. |
| HH coping well financially | 0.127 | -0.342 | 0.033 | 0.447 | 0.413 | 0.443 |
|  | (0.763) | (0.714) | (0.702) | (0.472) | (0.450) | (0.485) |
| baseline - single parent HH | ref. |  | ref. | ref. | ref. | ref. |
| Partner in HH | -1.150 | -1.727* | -1.163 | -1.584* | -0.087 | -1.460* |
|  | (1.467) | (0.834) | (0.972) | (0.761) | (1.055) | (0.581) |
| baseline - Not limited by disability | ref. | ref. | ref. | ref. | ref. | ref. |
| Limited a little | - | - | - | - | - | - |
|  | - | - | - | - | - | - |
| Limited a lot | - | - | - | - | - | - |
|  | - | - | - | - | - | - |
| baseline - White | ref. | ref. | ref. | ref. | ref. | ref. |
| Mixed | - | - | - | - | - | - |
|  | - | - | - | - | - | - |
| Asian | - | - | - | - | - | - |
|  | - | - | - | - | - | - |
| Other | - | - | - | - | - | - |
|  | - | - | - | - | - | - |
| Black | - | - | - | - | - | - |
|  | - | - | - | - | - | - |
| LA Economic disadvantage | - | - | - | - | - | - |
|  | - | - | - | - | - | - |
| LA Social disadvantage | - | - | - | - | - | - |
|  | - | - | - | - | - | - |
| LA Social disadvantage * LA Social disadvantage | - | - | - | - | - | - |
|  | - | - | - | - | - | - |
| baseline - Male | ref. | ref. | ref. | ref. | ref. | ref. |
| Female | - | - | - | - | - | - |
|  | - | - | - | - | - | - |
| LA COVID-19 case rate | 0.008 | 0.002 | -0.000 | 0.015* | 0.017* | 0.013* |
|  | (0.013) | (0.012) | (0.011) | (0.007) | (0.007) | (0.006) |
| baseline - Survey wave 2017-18 | ref. | ref. | ref. | ref. | ref. | ref. |
| November 2020 | 1.419*** | 1.411*** | 7.930*** | 0.829*** | 0.781*** | 4.259** |
|  | (0.281) | (0.257) | (1.874) | (0.242) | (0.227) | (1.378) |
| *Social capital measures* |  |  |  |  |  |  |
| Eating family meal (F) |  | -0.805* | -0.726* |  | -0.249 | -0.165 |
|  |  | (0.375) | (0.325) |  | (0.198) | (0.214) |
| November 2020 * Family meal (F) |  |  | -0.342 |  |  | -0.023 |
|  |  |  | (0.325) |  |  | (0.242) |
| Close friends (N) |  | -0.045+ | -0.030 |  | 0.004 | 0.014 |
|  |  | (0.026) | (0.021) |  | (0.018) | (0.013) |
| November 2020 * Close friends (N) |  |  | -0.122* |  |  | -0.042 |
|  |  |  | (0.051) |  |  | (0.034) |
| Family support |  | -0.461 | 0.879 |  | -0.053 | 0.081 |
|  |  | (0.694) | (0.752) |  | (0.496) | (0.412) |
| November 2020 * Family support |  |  | -1.474* |  |  | -0.412 |
|  |  |  | (0.616) |  |  | (0.441) |
| Neighbourhood safety |  | -0.339 | -0.156 |  | 0.253+ | 0.655** |
|  |  | (0.207) | (0.231) |  | (0.149) | (0.217) |
| November 2020 * Neigh. safety |  |  | -0.211 |  |  | -0.758* |
|  |  |  | (0.267) |  |  | (0.295) |
|  |  |  |  |  |  |  |
| Constant | 4.350** | 11.009*** | 5.195* | 5.285*** | 4.841** | 3.772** |
|  | (1.353) | (1.873) | (2.268) | (0.738) | (1.530) | (1.440) |
| Observations | 1214 | 1214 | 1214 | 1214 | 1214 | 1214 |

*Notes*: F=Frequency; N=Number; HH=Household; LA=Local Authority; SDQ=Strengths and Difficulties Questionnaire; Internalize=Internalizing scores; UK Household Longitudinal Study Mainstage and UK Household Longitudinal Study COVID-19 data; standard errors in parentheses.

+ p<.10, * p<.05, ** p<.01, *** p<.001

**Table S.6 – Testing the moderating role of social capital alongside interaction-terms between survey-period and all model covariates - internalising scores; pooled cross-sectional analysis**

|  | M1 | M2 | M3 | M4 | M5 | M6 | M7 | M8 | M9 | M10 | M11 | M12 |
| --- | --- | --- | --- | --- | --- | --- | --- | --- | --- | --- | --- | --- |
| SDQ type | Int. | Int. | Int. | Int. | Int. | Int. | Int. | Int. | Int. | Int. | Int. | Int. |
|  |  |  |  |  |  |  |  |  |  |  |  |  |
| baseline - No degree in HH | ref. | ref. | ref. | ref. | ref. | ref. | ref. | ref. | ref. | ref. | ref. | ref. |
| Degree in HH | -0.266 | -0.209 | -0.263 | -0.258 | -0.330+ | -0.242 | -0.267 | -0.265 | -0.260 | -0.264 | -0.275 | -0.264 |
|  | (0.181) | (0.296) | (0.181) | (0.181) | (0.179) | (0.183) | (0.183) | (0.182) | (0.181) | (0.182) | (0.180) | (0.182) |
| baseline - None employed in HH | ref. | ref. | ref. | ref. | ref. | ref. | ref. | ref. | ref. | ref. | ref. | ref. |
| 1 | 0.380 | 0.389 | 0.573 | 0.366 | 0.357 | 0.336 | 0.375 | 0.370 | 0.361 | 0.381 | 0.402 | 0.388 |
|  | (0.270) | (0.271) | (0.608) | (0.266) | (0.270) | (0.268) | (0.269) | (0.267) | (0.266) | (0.269) | (0.267) | (0.268) |
| 2+ | 0.243 | 0.236 | 0.447 | 0.211 | 0.179 | 0.231 | 0.247 | 0.229 | 0.247 | 0.243 | 0.261 | 0.252 |
|  | (0.296) | (0.295) | (0.626) | (0.295) | (0.290) | (0.295) | (0.296) | (0.294) | (0.293) | (0.296) | (0.292) | (0.294) |
| baseline - HH not coping financially | ref. | ref. | ref. | ref. | ref. | ref. | ref. | ref. | ref. | ref. | ref. | ref. |
| HH coping well financially | -0.178 | -0.181 | -0.178 | -0.188 | -0.149 | -0.206 | -0.189 | -0.158 | -0.190 | -0.191 | -0.168 | -0.178 |
|  | (0.214) | (0.211) | (0.215) | (0.358) | (0.210) | (0.212) | (0.216) | (0.212) | (0.212) | (0.214) | (0.214) | (0.213) |
| baseline - single parent HH | ref. | ref. | ref. | ref. | ref. | ref. | ref. | ref. | ref. | ref. | ref. | ref. |
| Partner in HH | 0.293 | 0.284 | 0.288 | 0.286 | 0.985* | 0.304 | 0.309 | 0.285 | 0.279 | 0.291 | 0.303 | 0.291 |
|  | (0.225) | (0.223) | (0.226) | (0.227) | (0.392) | (0.225) | (0.223) | (0.223) | (0.226) | (0.226) | (0.228) | (0.227) |
| baseline - Not limited by disability | ref. | ref. | ref. | ref. | ref. | ref. | ref. | ref. | ref. | ref. | ref. | ref. |
| Limited a little | 1.491*** | 1.487*** | 1.495*** | 1.499*** | 1.505*** | 2.214*** | 1.494*** | 1.493*** | 1.493*** | 1.486*** | 1.480*** | 1.488*** |
|  | (0.310) | (0.309) | (0.310) | (0.310) | (0.310) | (0.577) | (0.310) | (0.311) | (0.311) | (0.309) | (0.311) | (0.310) |
| Limited a lot | 2.256*** | 2.239*** | 2.247*** | 2.212*** | 2.279*** | 0.453 | 2.271*** | 2.290*** | 2.243*** | 2.245*** | 2.261*** | 2.256*** |
|  | (0.478) | (0.478) | (0.476) | (0.479) | (0.469) | (0.633) | (0.486) | (0.474) | (0.474) | (0.477) | (0.470) | (0.480) |
| baseline - White | ref. | ref. | ref. | ref. | ref. | ref. | ref. | ref. | ref. | ref. | ref. | ref. |
| Mixed | -0.591* | -0.605* | -0.579* | -0.574* | -0.586* | -0.610* | -0.895* | -0.595* | -0.588* | -0.599* | -0.624* | -0.599* |
|  | (0.278) | (0.279) | (0.279) | (0.281) | (0.285) | (0.279) | (0.438) | (0.279) | (0.280) | (0.278) | (0.278) | (0.278) |
| Asian | -0.974*** | -0.977*** | -0.957** | -0.969** | -0.965** | -1.007*** | -0.853* | -0.964*** | -0.976*** | -0.970** | -0.971*** | -0.971*** |
|  | (0.295) | (0.294) | (0.293) | (0.297) | (0.296) | (0.301) | (0.406) | (0.292) | (0.295) | (0.296) | (0.294) | (0.295) |
| Other | 0.197 | 0.234 | 0.222 | 0.223 | 0.290 | 0.288 | 0.414 | 0.182 | 0.185 | 0.179 | 0.192 | 0.214 |
|  | (0.573) | (0.565) | (0.572) | (0.581) | (0.567) | (0.565) | (0.847) | (0.573) | (0.580) | (0.572) | (0.583) | (0.576) |
| Black | -0.820 | -0.808 | -0.787 | -0.777 | -0.781 | -0.810 | -0.753 | -0.846 | -0.823 | -0.808 | -0.799 | -0.814 |
|  | (0.536) | (0.537) | (0.541) | (0.550) | (0.536) | (0.550) | (0.537) | (0.536) | (0.531) | (0.538) | (0.541) | (0.535) |
| baseline - Male | ref. | ref. | ref. | ref. | ref. | ref. | ref. | ref. | ref. | ref. | ref. | ref. |
| Female | 0.790*** | 0.792*** | 0.788*** | 0.794*** | 0.789*** | 0.806*** | 0.790*** | 0.331 | 0.784*** | 0.791*** | 0.789*** | 0.789*** |
|  | (0.156) | (0.156) | (0.155) | (0.155) | (0.156) | (0.157) | (0.155) | (0.292) | (0.156) | (0.155) | (0.156) | (0.155) |
| baseline - Aged 10-11 | ref. | ref. | ref. | ref. | ref. | ref. | ref. | ref. | ref. | ref. | ref. | ref. |
| 12-13 | 0.077 | 0.079 | 0.073 | 0.060 | 0.092 | 0.095 | 0.069 | 0.089 | 0.108 | 0.079 | 0.084 | 0.077 |
|  | (0.173) | (0.173) | (0.173) | (0.174) | (0.171) | (0.171) | (0.173) | (0.174) | (0.285) | (0.172) | (0.173) | (0.173) |
| 14-15 | 0.585** | 0.587** | 0.581** | 0.573** | 0.603** | 0.587** | 0.582** | 0.584** | 1.049 | 0.585** | 0.579** | 0.581** |
|  | (0.201) | (0.202) | (0.202) | (0.202) | (0.201) | (0.196) | (0.202) | (0.202) | (1.315) | (0.201) | (0.200) | (0.201) |
| LA Economic disadvantage | -0.103 | -0.102 | -0.102 | -0.109 | -0.077 | -0.106 | -0.107 | -0.110 | -0.105 | -0.189 | -0.126 | -0.106 |
|  | (0.160) | (0.159) | (0.162) | (0.160) | (0.161) | (0.162) | (0.162) | (0.158) | (0.160) | (0.229) | (0.161) | (0.160) |
| LA Social disadvantage | 0.285+ | 0.275+ | 0.286+ | 0.281+ | 0.290+ | 0.273+ | 0.287+ | 0.297* | 0.284+ | 0.292+ | 0.538* | 0.282+ |
|  | (0.152) | (0.151) | (0.152) | (0.152) | (0.153) | (0.152) | (0.152) | (0.151) | (0.151) | (0.152) | (0.240) | (0.152) |
| LA Social disadvantage * LA Social disadvantage | 0.147* | 0.144* | 0.148* | 0.148* | 0.146* | 0.145* | 0.146* | 0.153* | 0.147* | 0.147* | 0.188* | 0.147* |
|  | (0.062) | (0.062) | (0.062) | (0.063) | (0.062) | (0.060) | (0.062) | (0.063) | (0.062) | (0.063) | (0.092) | (0.062) |
| LA COVID-19 case rate | -0.000 | -0.000 | -0.000 | -0.000 | -0.000 | -0.000 | -0.000 | -0.000 | -0.000 | -0.000 | -0.000 | 0.002 |
|  | (0.000) | (0.000) | (0.000) | (0.000) | (0.000) | (0.000) | (0.000) | (0.000) | (0.000) | (0.000) | (0.000) | (0.003) |
| baseline - Survey wave 2017-18 | ref. | ref. | ref. | ref. | ref. | ref. | ref. | ref. | ref. | ref. | ref. | ref. |
| August 2020 | 5.729*** | 5.753*** | 5.979*** | 5.766*** | 5.871*** | 6.137*** | 5.738*** | 5.371*** | 6.045*** | 4.576** | 7.772*** | 5.710*** |
|  | (1.425) | (1.422) | (1.460) | (1.425) | (1.422) | (1.500) | (1.482) | (1.459) | (1.448) | (1.722) | (1.788) | (1.422) |
| November 2020 | 8.708*** | 8.619*** | 8.824*** | 8.705*** | 9.152*** | 8.864*** | 8.872*** | 8.134*** | 8.531*** | 8.077*** | 9.892*** | 8.614*** |
|  | (1.589) | (1.591) | (1.693) | (1.579) | (1.580) | (1.645) | (1.653) | (1.566) | (1.619) | (1.784) | (1.812) | (1.571) |
| March 2021 | 7.148*** | 7.225*** | 7.184*** | 7.183*** | 7.185*** | 7.292*** | 7.208*** | 6.710*** | 7.339*** | 7.699*** | 8.280*** | 7.166*** |
|  | (1.525) | (1.540) | (1.562) | (1.518) | (1.531) | (1.733) | (1.594) | (1.493) | (1.538) | (1.747) | (1.853) | (1.526) |
| *Social capital measures* |  |  |  |  |  |  |  |  |  |  |  |  |
| Eating family meal (F) | 0.017 | 0.015 | 0.010 | 0.017 | 0.004 | -0.032 | 0.019 | 0.022 | 0.019 | 0.013 | 0.023 | 0.017 |
|  | (0.177) | (0.180) | (0.180) | (0.178) | (0.177) | (0.167) | (0.179) | (0.176) | (0.176) | (0.177) | (0.178) | (0.177) |
| August 2020 * Eating family meal (F) | -0.126 | -0.132 | -0.112 | -0.149 | -0.092 | -0.024 | -0.125 | -0.132 | -0.132 | -0.125 | -0.135 | -0.126 |
|  | (0.205) | (0.209) | (0.205) | (0.207) | (0.205) | (0.190) | (0.205) | (0.203) | (0.205) | (0.206) | (0.204) | (0.205) |
| November 2020 * Eating family meal (F) | -0.190 | -0.189 | -0.186 | -0.187 | -0.155 | -0.122 | -0.202 | -0.184 | -0.184 | -0.188 | -0.199 | -0.197 |
|  | (0.201) | (0.204) | (0.205) | (0.202) | (0.202) | (0.191) | (0.207) | (0.201) | (0.198) | (0.201) | (0.202) | (0.201) |
| March 2021 * Eating family meal (F) | -0.447* | -0.441* | -0.443* | -0.427* | -0.417* | -0.387+ | -0.466* | -0.453* | -0.460* | -0.444* | -0.453* | -0.449* |
|  | (0.203) | (0.206) | (0.207) | (0.205) | (0.203) | (0.199) | (0.208) | (0.202) | (0.203) | (0.202) | (0.205) | (0.203) |
| Close friends (N) | -0.009 | -0.009 | -0.009 | -0.009 | -0.005 | -0.016 | -0.009 | -0.012 | -0.008 | -0.009 | -0.010 | -0.009 |
|  | (0.040) | (0.040) | (0.040) | (0.040) | (0.039) | (0.039) | (0.040) | (0.039) | (0.040) | (0.040) | (0.040) | (0.040) |
| August 2020 * Close friends (N) | -0.094* | -0.095* | -0.096* | -0.096* | -0.099* | -0.084* | -0.092* | -0.091* | -0.095* | -0.094* | -0.093* | -0.094* |
|  | (0.039) | (0.039) | (0.039) | (0.039) | (0.039) | (0.039) | (0.039) | (0.038) | (0.039) | (0.039) | (0.039) | (0.039) |
| November 2020 * Close friends (N) | -0.082+ | -0.081+ | -0.083+ | -0.083+ | -0.086* | -0.073+ | -0.084+ | -0.079+ | -0.083+ | -0.083+ | -0.082+ | -0.081+ |
|  | (0.043) | (0.043) | (0.043) | (0.043) | (0.042) | (0.042) | (0.043) | (0.042) | (0.043) | (0.043) | (0.042) | (0.043) |
| March 2021 * Close friends (N) | -0.092* | -0.092* | -0.093* | -0.091* | -0.096* | -0.083+ | -0.094* | -0.089* | -0.093* | -0.093* | -0.092* | -0.092* |
|  | (0.045) | (0.045) | (0.044) | (0.044) | (0.045) | (0.044) | (0.045) | (0.044) | (0.045) | (0.044) | (0.044) | (0.045) |
| Family support | -0.735* | -0.733* | -0.738* | -0.741* | -0.807* | -0.616+ | -0.737* | -0.724* | -0.747* | -0.725* | -0.712* | -0.742* |
|  | (0.359) | (0.359) | (0.364) | (0.366) | (0.365) | (0.352) | (0.360) | (0.359) | (0.356) | (0.360) | (0.356) | (0.359) |
| August 2020 * Family support | -0.818* | -0.810* | -0.765+ | -0.836* | -0.671+ | -1.028** | -0.831* | -0.817* | -0.865* | -0.845* | -0.862* | -0.810* |
|  | (0.401) | (0.402) | (0.399) | (0.404) | (0.400) | (0.394) | (0.404) | (0.402) | (0.402) | (0.406) | (0.396) | (0.401) |
| November 2020 * Family support | -1.438*** | -1.436*** | -1.446*** | -1.424*** | -1.302*** | -1.570*** | -1.429*** | -1.453*** | -1.392*** | -1.451*** | -1.463*** | -1.422*** |
|  | (0.392) | (0.392) | (0.396) | (0.393) | (0.388) | (0.401) | (0.394) | (0.398) | (0.390) | (0.395) | (0.388) | (0.392) |
| March 2021 * Family support | -0.453 | -0.468 | -0.459 | -0.431 | -0.357 | -0.570 | -0.436 | -0.450 | -0.478 | -0.460 | -0.479 | -0.452 |
|  | (0.479) | (0.483) | (0.486) | (0.487) | (0.484) | (0.506) | (0.481) | (0.481) | (0.478) | (0.479) | (0.476) | (0.480) |
| Friend support | -0.499 | -0.504 | -0.504 | -0.493 | -0.542 | -0.481 | -0.507 | -0.495 | -0.498 | -0.507 | -0.512 | -0.496 |
|  | (0.331) | (0.330) | (0.331) | (0.331) | (0.330) | (0.321) | (0.330) | (0.330) | (0.330) | (0.331) | (0.330) | (0.331) |
| August 2020 * Friend support | -0.666* | -0.663* | -0.656* | -0.705* | -0.655* | -0.770* | -0.678* | -0.678* | -0.640+ | -0.655+ | -0.659* | -0.667* |
|  | (0.336) | (0.335) | (0.333) | (0.333) | (0.328) | (0.320) | (0.332) | (0.339) | (0.330) | (0.336) | (0.333) | (0.337) |
| November 2020 * Friend support | -0.961** | -0.964** | -0.953** | -0.962** | -0.930** | -1.003** | -0.944** | -0.979** | -0.975** | -0.951** | -0.949** | -0.966** |
|  | (0.343) | (0.343) | (0.340) | (0.341) | (0.336) | (0.336) | (0.344) | (0.349) | (0.344) | (0.344) | (0.343) | (0.345) |
| March 2021 * Friend support | -0.951* | -0.953* | -0.961* | -0.960** | -0.919* | -0.992** | -0.940* | -0.956* | -0.937* | -0.937* | -0.938* | -0.956* |
|  | (0.374) | (0.374) | (0.380) | (0.370) | (0.373) | (0.358) | (0.374) | (0.378) | (0.374) | (0.373) | (0.373) | (0.375) |
| Neighbourhood safety | -0.456* | -0.453* | -0.455* | -0.452* | -0.436* | -0.435* | -0.444* | -0.563** | -0.453* | -0.458* | -0.453* | -0.456* |
|  | (0.205) | (0.204) | (0.205) | (0.204) | (0.201) | (0.200) | (0.209) | (0.213) | (0.206) | (0.204) | (0.207) | (0.205) |
| August 2020 * Neighbourhood safety | -0.153 | -0.161 | -0.165 | -0.160 | -0.172 | -0.170 | -0.173 | -0.063 | -0.120 | -0.149 | -0.164 | -0.152 |
|  | (0.215) | (0.215) | (0.214) | (0.214) | (0.210) | (0.209) | (0.218) | (0.229) | (0.213) | (0.215) | (0.218) | (0.215) |
| November 2020 * Neighbourhood safety | -0.168 | -0.162 | -0.171 | -0.164 | -0.188 | -0.184 | -0.186 | 0.003 | -0.203 | -0.167 | -0.175 | -0.176 |
|  | (0.214) | (0.214) | (0.214) | (0.213) | (0.208) | (0.214) | (0.219) | (0.229) | (0.216) | (0.214) | (0.216) | (0.214) |
| March 2021 * Neighbourhood safety | -0.190 | -0.196 | -0.190 | -0.206 | -0.217 | -0.206 | -0.193 | -0.078 | -0.171 | -0.186 | -0.195 | -0.191 |
|  | (0.245) | (0.245) | (0.244) | (0.244) | (0.239) | (0.235) | (0.249) | (0.254) | (0.247) | (0.243) | (0.247) | (0.245) |
| HH social capital | -0.055 | -0.060 | -0.059 | -0.056 | -0.076 | -0.059 | -0.051 | -0.064 | -0.058 | -0.061 | -0.046 | -0.056 |
|  | (0.093) | (0.094) | (0.094) | (0.093) | (0.095) | (0.096) | (0.097) | (0.090) | (0.092) | (0.095) | (0.093) | (0.093) |
| August 2020 * HH Social capital | 0.036 | 0.036 | 0.047 | 0.007 | 0.072 | 0.023 | 0.042 | 0.039 | 0.055 | 0.026 | 0.024 | 0.036 |
|  | (0.213) | (0.217) | (0.209) | (0.213) | (0.207) | (0.215) | (0.215) | (0.213) | (0.214) | (0.214) | (0.214) | (0.213) |
| November 2020 * HH Social capital | 0.106 | 0.112 | 0.109 | 0.118 | 0.214 | 0.100 | 0.094 | 0.071 | 0.102 | 0.103 | 0.100 | 0.103 |
|  | (0.226) | (0.224) | (0.225) | (0.225) | (0.216) | (0.228) | (0.231) | (0.227) | (0.226) | (0.227) | (0.226) | (0.226) |
| March 2021 * HH Social capital | -0.008 | -0.002 | 0.002 | 0.005 | -0.006 | -0.013 | -0.033 | -0.011 | 0.009 | 0.007 | -0.009 | -0.005 |
|  | (0.226) | (0.224) | (0.224) | (0.228) | (0.224) | (0.225) | (0.233) | (0.227) | (0.229) | (0.227) | (0.226) | (0.226) |
| LA Relationships Index | -0.159 | -0.164 | -0.165 | -0.170 | -0.116 | -0.211 | -0.155 | -0.132 | -0.161 | -0.219 | 0.005 | -0.165 |
|  | (0.270) | (0.269) | (0.270) | (0.272) | (0.277) | (0.271) | (0.273) | (0.267) | (0.270) | (0.304) | (0.297) | (0.269) |
| August 2020 * LA Relationships Index | 0.010 | 0.011 | 0.038 | -0.025 | -0.005 | 0.077 | 0.021 | -0.008 | 0.006 | 0.365 | -0.476 | 0.011 |
|  | (0.227) | (0.226) | (0.221) | (0.230) | (0.226) | (0.222) | (0.234) | (0.224) | (0.227) | (0.344) | (0.330) | (0.226) |
| November 2020 * LA Relationships Index | -0.096 | -0.075 | -0.091 | -0.086 | -0.113 | -0.054 | -0.116 | -0.135 | -0.094 | 0.090 | -0.378 | -0.093 |
|  | (0.208) | (0.209) | (0.207) | (0.208) | (0.204) | (0.206) | (0.214) | (0.208) | (0.208) | (0.297) | (0.319) | (0.207) |
| March 2021 * LA Relationships Index | -0.025 | -0.028 | -0.022 | -0.007 | -0.053 | 0.011 | -0.021 | -0.052 | -0.029 | -0.210 | -0.297 | -0.025 |
|  | (0.190) | (0.193) | (0.190) | (0.195) | (0.191) | (0.186) | (0.198) | (0.190) | (0.190) | (0.300) | (0.319) | (0.190) |
| Extra-curricular (N) | 0.257 | 0.255 | 0.257 | 0.256 | 0.225 | 0.247 | 0.247 | 0.241 | 0.253 | 0.263 | 0.287 | 0.256 |
|  | (0.183) | (0.185) | (0.183) | (0.183) | (0.186) | (0.177) | (0.183) | (0.180) | (0.184) | (0.183) | (0.184) | (0.183) |
| August 2020 * Extra-curricular (N) | -0.003 | 0.002 | 0.016 | -0.008 | 0.050 | -0.020 | 0.008 | 0.014 | -0.037 | -0.023 | -0.040 | -0.004 |
|  | (0.202) | (0.206) | (0.205) | (0.202) | (0.209) | (0.194) | (0.203) | (0.201) | (0.203) | (0.201) | (0.203) | (0.202) |
| November 2020 * Extra-curricular (N) | -0.106 | -0.105 | -0.106 | -0.103 | -0.035 | -0.103 | -0.086 | -0.098 | -0.072 | -0.118 | -0.135 | -0.111 |
|  | (0.208) | (0.211) | (0.209) | (0.208) | (0.213) | (0.203) | (0.209) | (0.207) | (0.210) | (0.209) | (0.207) | (0.209) |
| March 2021 *Extra-curricular (N) | -0.145 | -0.142 | -0.151 | -0.136 | -0.120 | -0.129 | -0.145 | -0.128 | -0.166 | -0.131 | -0.172 | -0.147 |
|  | (0.221) | (0.222) | (0.221) | (0.221) | (0.224) | (0.216) | (0.221) | (0.218) | (0.224) | (0.221) | (0.222) | (0.221) |
| August 2020 * Degree in HH |  | 0.010 |  |  |  |  |  |  |  |  |  |  |
|  |  | (0.342) |  |  |  |  |  |  |  |  |  |  |
| November 2020 * Degree in HH |  | -0.134 |  |  |  |  |  |  |  |  |  |  |
|  |  | (0.351) |  |  |  |  |  |  |  |  |  |  |
| March 2021 * Degree in HH |  | -0.083 |  |  |  |  |  |  |  |  |  |  |
|  |  | (0.352) |  |  |  |  |  |  |  |  |  |  |
| August 2020 * 1 employed in HH |  |  | -0.611 |  |  |  |  |  |  |  |  |  |
|  |  |  | (0.730) |  |  |  |  |  |  |  |  |  |
| August 2020 * 2+ employed in HH |  |  | -0.777 |  |  |  |  |  |  |  |  |  |
|  |  |  | (0.722) |  |  |  |  |  |  |  |  |  |
| November 2020 * 1 employed in HH |  |  | -0.101 |  |  |  |  |  |  |  |  |  |
|  |  |  | (0.749) |  |  |  |  |  |  |  |  |  |
| November 2020 * 2+ employed in HH |  |  | -0.085 |  |  |  |  |  |  |  |  |  |
|  |  |  | (0.759) |  |  |  |  |  |  |  |  |  |
| March 2021 * 1 employed in HH |  |  | -0.023 |  |  |  |  |  |  |  |  |  |
|  |  |  | (0.691) |  |  |  |  |  |  |  |  |  |
| March 2021 * 2+ employed in HH |  |  | 0.001 |  |  |  |  |  |  |  |  |  |
|  |  |  | (0.715) |  |  |  |  |  |  |  |  |  |
| August 2020 * HH coping well financially |  |  |  | 0.534 |  |  |  |  |  |  |  |  |
|  |  |  |  | (0.412) |  |  |  |  |  |  |  |  |
| November 2020 * HH coping well financially |  |  |  | -0.122 |  |  |  |  |  |  |  |  |
|  |  |  |  | (0.390) |  |  |  |  |  |  |  |  |
| March 2021 * HH coping well financially |  |  |  | -0.276 |  |  |  |  |  |  |  |  |
|  |  |  |  | (0.489) |  |  |  |  |  |  |  |  |
| August 2020 * Partner in HH |  |  |  |  | -0.966* |  |  |  |  |  |  |  |
|  |  |  |  |  | (0.446) |  |  |  |  |  |  |  |
| November 2020 * Partner in HH |  |  |  |  | -1.613*** |  |  |  |  |  |  |  |
|  |  |  |  |  | (0.440) |  |  |  |  |  |  |  |
| March 2021 * Partner in HH |  |  |  |  | -0.369 |  |  |  |  |  |  |  |
|  |  |  |  |  | (0.424) |  |  |  |  |  |  |  |
| August 2020 * Health limits a little |  |  |  |  |  | -1.470* |  |  |  |  |  |  |
|  |  |  |  |  |  | (0.583) |  |  |  |  |  |  |
| August 2020 * Health limits a lot |  |  |  |  |  | 3.407*** |  |  |  |  |  |  |
|  |  |  |  |  |  | (0.631) |  |  |  |  |  |  |
| November 2020 * Health limits a little |  |  |  |  |  | -0.866 |  |  |  |  |  |  |
|  |  |  |  |  |  | (0.634) |  |  |  |  |  |  |
| November 2020 * Health limits a lot |  |  |  |  |  | 2.458* |  |  |  |  |  |  |
|  |  |  |  |  |  | (1.033) |  |  |  |  |  |  |
| March 2021 * Health limits a little |  |  |  |  |  | -0.704 |  |  |  |  |  |  |
|  |  |  |  |  |  | (0.718) |  |  |  |  |  |  |
| March 2021 * Health limits a lot |  |  |  |  |  | 2.072* |  |  |  |  |  |  |
|  |  |  |  |  |  | (0.961) |  |  |  |  |  |  |
| August 2020 * Ethnic Mixed |  |  |  |  |  |  | 0.618 |  |  |  |  |  |
|  |  |  |  |  |  |  | (0.674) |  |  |  |  |  |
| August 2020 * Ethnic Asian |  |  |  |  |  |  | -0.231 |  |  |  |  |  |
|  |  |  |  |  |  |  | (0.599) |  |  |  |  |  |
| August 2020 * Ethnic Other |  |  |  |  |  |  | -0.768 |  |  |  |  |  |
|  |  |  |  |  |  |  | (0.847) |  |  |  |  |  |
| August 2020 * Ethnic Black |  |  |  |  |  |  | 0.941+ |  |  |  |  |  |
|  |  |  |  |  |  |  | (0.566) |  |  |  |  |  |
| November 2020 * Mixed |  |  |  |  |  |  | 0.157 |  |  |  |  |  |
|  |  |  |  |  |  |  | (0.604) |  |  |  |  |  |
| November 2020 * Ethnic Asian |  |  |  |  |  |  | -0.386 |  |  |  |  |  |
|  |  |  |  |  |  |  | (0.453) |  |  |  |  |  |
| November 2020 * Ethnic Other |  |  |  |  |  |  | -0.278 |  |  |  |  |  |
|  |  |  |  |  |  |  | (1.085) |  |  |  |  |  |
| November 2020 * Ethnic Black |  |  |  |  |  |  | -0.649 |  |  |  |  |  |
|  |  |  |  |  |  |  | (0.651) |  |  |  |  |  |
| March 2021 * Ethnic Mixed |  |  |  |  |  |  | 0.665 |  |  |  |  |  |
|  |  |  |  |  |  |  | (0.627) |  |  |  |  |  |
| March 2021 * Ethnic Asian |  |  |  |  |  |  | 0.192 |  |  |  |  |  |
|  |  |  |  |  |  |  | (0.516) |  |  |  |  |  |
| March 2021 * Ethnic Other |  |  |  |  |  |  | 0.328 |  |  |  |  |  |
|  |  |  |  |  |  |  | (1.156) |  |  |  |  |  |
| March 2021 * Ethnic Black |  |  |  |  |  |  | -0.542 |  |  |  |  |  |
|  |  |  |  |  |  |  | (0.791) |  |  |  |  |  |
| August 2020 * Female |  |  |  |  |  |  |  | 0.361 |  |  |  |  |
|  |  |  |  |  |  |  |  | (0.325) |  |  |  |  |
| November 2020 * Female |  |  |  |  |  |  |  | 0.823* |  |  |  |  |
|  |  |  |  |  |  |  |  | (0.343) |  |  |  |  |
| March 2021 * Female |  |  |  |  |  |  |  | 0.472 |  |  |  |  |
|  |  |  |  |  |  |  |  | (0.326) |  |  |  |  |
| August 2020 * Age 12-13 |  |  |  |  |  |  |  |  | -0.450 |  |  |  |
|  |  |  |  |  |  |  |  |  | (0.391) |  |  |  |
| August 2020 * Age 14-15 |  |  |  |  |  |  |  |  | -0.971 |  |  |  |
|  |  |  |  |  |  |  |  |  | (1.341) |  |  |  |
| November 2020 * Age 12-13 |  |  |  |  |  |  |  |  | 0.184 |  |  |  |
|  |  |  |  |  |  |  |  |  | (0.395) |  |  |  |
| November 2020 * Age 14-15 |  |  |  |  |  |  |  |  | -0.109 |  |  |  |
|  |  |  |  |  |  |  |  |  | (1.324) |  |  |  |
| March 2021 * Age 12-13 |  |  |  |  |  |  |  |  | -0.206 |  |  |  |
|  |  |  |  |  |  |  |  |  | (0.469) |  |  |  |
| March 2021 * Age 14-15 |  |  |  |  |  |  |  |  | -0.752 |  |  |  |
|  |  |  |  |  |  |  |  |  | (1.337) |  |  |  |
| August 2020 * LA Economic disadvantage |  |  |  |  |  |  |  |  |  | 0.433 |  |  |
|  |  |  |  |  |  |  |  |  |  | (0.316) |  |  |
| November 2020 * LA Economic disadvantage |  |  |  |  |  |  |  |  |  | 0.232 |  |  |
|  |  |  |  |  |  |  |  |  |  | (0.252) |  |  |
| March 2021 * LA Economic disadvantage |  |  |  |  |  |  |  |  |  | -0.216 |  |  |
|  |  |  |  |  |  |  |  |  |  | (0.286) |  |  |
| August 2020 * LA Social disadvantage |  |  |  |  |  |  |  |  |  |  | -0.665* |  |
|  |  |  |  |  |  |  |  |  |  |  | (0.311) |  |
| November 2020 * LA Social disadvantage |  |  |  |  |  |  |  |  |  |  | -0.382 |  |
|  |  |  |  |  |  |  |  |  |  |  | (0.320) |  |
| March 2021 * LA Social disadvantage |  |  |  |  |  |  |  |  |  |  | -0.391 |  |
|  |  |  |  |  |  |  |  |  |  |  | (0.349) |  |
| August 2020 * LA Social disadvantage-sq. |  |  |  |  |  |  |  |  |  |  | -0.079 |  |
|  |  |  |  |  |  |  |  |  |  |  | (0.098) |  |
| November 2020 * LA Social disadvantage-sq. |  |  |  |  |  |  |  |  |  |  | -0.056 |  |
|  |  |  |  |  |  |  |  |  |  |  | (0.115) |  |
| March 2021 * LA Social disadvantage-sq. |  |  |  |  |  |  |  |  |  |  | -0.086 |  |
|  |  |  |  |  |  |  |  |  |  |  | (0.106) |  |
| August 2020 * LA COVID-19 case rate |  |  |  |  |  |  |  |  |  |  |  | -0.002 |
|  |  |  |  |  |  |  |  |  |  |  |  | (0.003) |
| November 2020 * LA COVID-19 case rate |  |  |  |  |  |  |  |  |  |  |  | -0.007 |
|  |  |  |  |  |  |  |  |  |  |  |  | (0.006) |
| March 2021 * LA COVID-19 case rate |  |  |  |  |  |  |  |  |  |  |  | 0.000 |
|  |  |  |  |  |  |  |  |  |  |  |  |  |
| Constant | 8.906*** | 8.937*** | 8.785*** | 8.968*** | 8.601*** | 8.852*** | 8.862*** | 9.326*** | 8.919*** | 9.150*** | 8.147*** | 8.930*** |
|  | (1.509) | (1.507) | (1.562) | (1.513) | (1.500) | (1.567) | (1.536) | (1.479) | (1.517) | (1.614) | (1.581) | (1.502) |
| Observations | 3176 | 3176 | 3176 | 3176 | 3176 | 3176 | 3176 | 3176 | 3176 | 3176 | 3176 | 3176 |

*Notes*: F=Frequency; N=Number; HH=Household; LA=Local Authority; SDQ=Strengths and Difficulties Questionnaire; Int.=Internalizing scores; Sq.=squared; UK Household Longitudinal Study Mainstage and UK Household Longitudinal Study COVID-19 data; standard errors in parentheses.

+ p<.10, * p<.05, ** p<.01, *** p<.001

**Table S.7 – Testing the moderating role of social capital alongside interaction-terms between survey-period and all model covariates - externalising scores; pooled cross-sectional analysis**

|  | M1 | M2 | M3 | M4 | M5 | M6 | M7 | M8 | M9 | M10 | M11 | M12 |
| --- | --- | --- | --- | --- | --- | --- | --- | --- | --- | --- | --- | --- |
| SDQ type | Ext. | Ext. | Ext. | Ext. | Ext. | Ext. | Ext. | Ext. | Ext. | Ext. | Ext. | Ext. |
|  |  |  |  |  |  |  |  |  |  |  |  |  |
| baseline - No degree in HH | ref. | ref. | ref. | ref. | ref. | ref. | ref. | ref. | ref. | ref. | ref. | ref. |
| Degree in HH | -0.478* | -0.586* | -0.476* | -0.484* | -0.492* | -0.470* | -0.461* | -0.473* | -0.478* | -0.476* | -0.481* | -0.479* |
|  | (0.204) | (0.275) | (0.205) | (0.206) | (0.210) | (0.206) | (0.205) | (0.203) | (0.204) | (0.204) | (0.206) | (0.204) |
| baseline - None employed in HH | ref. | ref. | ref. | ref. | ref. | ref. | ref. | ref. | ref. | ref. | ref. | ref. |
| 1 | 0.497* | 0.504* | 0.324 | 0.501* | 0.503* | 0.532* | 0.495* | 0.475+ | 0.486+ | 0.500* | 0.515* | 0.490* |
|  | (0.247) | (0.250) | (0.733) | (0.245) | (0.245) | (0.259) | (0.244) | (0.245) | (0.249) | (0.247) | (0.245) | (0.248) |
| 2+ | 0.330 | 0.326 | 0.302 | 0.319 | 0.313 | 0.348 | 0.327 | 0.309 | 0.334 | 0.334 | 0.343 | 0.326 |
|  | (0.267) | (0.271) | (0.694) | (0.268) | (0.264) | (0.275) | (0.265) | (0.266) | (0.268) | (0.267) | (0.264) | (0.267) |
| baseline - HH not coping financially | ref. | ref. | ref. | ref. | ref. | ref. | ref. | ref. | ref. | ref. | ref. | ref. |
| HH coping well financially | -0.141 | -0.156 | -0.147 | -0.276 | -0.139 | -0.109 | -0.136 | -0.128 | -0.143 | -0.144 | -0.131 | -0.138 |
|  | (0.191) | (0.191) | (0.185) | (0.344) | (0.191) | (0.191) | (0.191) | (0.188) | (0.189) | (0.191) | (0.192) | (0.191) |
| baseline - single parent HH | ref. | ref. | ref. | ref. | ref. | ref. | ref. | ref. | ref. | ref. | ref. | ref. |
| Partner in HH | 0.309 | 0.316 | 0.312 | 0.319 | 0.459 | 0.304 | 0.295 | 0.296 | 0.313 | 0.310 | 0.321 | 0.310 |
|  | (0.207) | (0.203) | (0.206) | (0.207) | (0.379) | (0.207) | (0.205) | (0.206) | (0.207) | (0.207) | (0.205) | (0.206) |
| baseline - Not limited by disability | ref. | ref. | ref. | ref. | ref. | ref. | ref. | ref. | ref. | ref. | ref. | ref. |
| Limited a little | 1.068** | 1.063** | 1.077** | 1.075** | 1.075** | 0.957* | 1.064** | 1.075** | 1.072** | 1.067** | 1.053** | 1.070** |
|  | (0.341) | (0.341) | (0.341) | (0.343) | (0.342) | (0.485) | (0.342) | (0.342) | (0.340) | (0.342) | (0.342) | (0.342) |
| Limited a lot | 2.392*** | 2.374*** | 2.382*** | 2.375*** | 2.394*** | 1.695** | 2.420*** | 2.422*** | 2.372*** | 2.384*** | 2.393*** | 2.389*** |
|  | (0.599) | (0.600) | (0.607) | (0.603) | (0.606) | (0.657) | (0.604) | (0.614) | (0.606) | (0.604) | (0.599) | (0.598) |
| baseline - White | ref. | ref. | ref. | ref. | ref. | ref. | ref. | ref. | ref. | ref. | ref. | ref. |
| Mixed | -0.040 | -0.034 | -0.010 | -0.018 | -0.014 | -0.054 | -0.675 | -0.041 | -0.023 | -0.044 | -0.054 | -0.038 |
|  | (0.320) | (0.319) | (0.319) | (0.320) | (0.319) | (0.322) | (0.431) | (0.320) | (0.320) | (0.323) | (0.322) | (0.320) |
| Asian | -0.698* | -0.693* | -0.680* | -0.684* | -0.681* | -0.713* | -0.735* | -0.691* | -0.712* | -0.696* | -0.696* | -0.697* |
|  | (0.307) | (0.307) | (0.306) | (0.305) | (0.306) | (0.308) | (0.358) | (0.304) | (0.310) | (0.307) | (0.305) | (0.307) |
| Other | 0.019 | 0.032 | 0.043 | 0.018 | 0.052 | 0.048 | -0.678 | 0.007 | 0.006 | 0.009 | 0.031 | 0.016 |
|  | (0.640) | (0.638) | (0.636) | (0.636) | (0.634) | (0.634) | (1.167) | (0.642) | (0.642) | (0.642) | (0.639) | (0.641) |
| Black | -0.633 | -0.628 | -0.615 | -0.616 | -0.619 | -0.648 | -0.693 | -0.663 | -0.637 | -0.628 | -0.617 | -0.635 |
|  | (0.602) | (0.603) | (0.607) | (0.608) | (0.604) | (0.607) | (0.790) | (0.600) | (0.608) | (0.606) | (0.610) | (0.600) |
| baseline - Male | ref. | ref. | ref. | ref. | ref. | ref. | ref. | ref. | ref. | ref. | ref. | ref. |
| Female | -0.591** | -0.585** | -0.588** | -0.588** | -0.591** | -0.590** | -0.593** | -0.995** | -0.589** | -0.592** | -0.599** | -0.591** |
|  | (0.187) | (0.187) | (0.189) | (0.187) | (0.187) | (0.189) | (0.187) | (0.318) | (0.188) | (0.188) | (0.188) | (0.187) |
| baseline - Aged 10-11 | ref. | ref. | ref. | ref. | ref. | ref. | ref. | ref. | ref. | ref. | ref. | ref. |
| 12-13 | 0.019 | 0.020 | 0.021 | 0.011 | 0.023 | 0.023 | 0.017 | 0.031 | 0.272 | 0.021 | 0.031 | 0.018 |
|  | (0.185) | (0.185) | (0.186) | (0.186) | (0.184) | (0.185) | (0.184) | (0.187) | (0.280) | (0.185) | (0.184) | (0.185) |
| 14-15 | 0.230 | 0.237 | 0.248 | 0.226 | 0.237 | 0.234 | 0.244 | 0.234 | 1.100 | 0.232 | 0.221 | 0.230 |
|  | (0.236) | (0.236) | (0.235) | (0.236) | (0.236) | (0.235) | (0.235) | (0.235) | (0.713) | (0.235) | (0.234) | (0.235) |
| LA Economic disadvantage | -0.229 | -0.233 | -0.230 | -0.232 | -0.227 | -0.233 | -0.235 | -0.234 | -0.234 | -0.258 | -0.250 | -0.228 |
|  | (0.182) | (0.181) | (0.181) | (0.181) | (0.182) | (0.182) | (0.180) | (0.181) | (0.182) | (0.243) | (0.183) | (0.182) |
| LA Social disadvantage | 0.254 | 0.255 | 0.258 | 0.255 | 0.257 | 0.249 | 0.248 | 0.266 | 0.256 | 0.257 | 0.524* | 0.254 |
|  | (0.185) | (0.184) | (0.184) | (0.184) | (0.184) | (0.185) | (0.183) | (0.185) | (0.184) | (0.185) | (0.246) | (0.186) |
| LA Social disadvantage * LA Social disadvantage | 0.107 | 0.107 | 0.109 | 0.108 | 0.106 | 0.106 | 0.104 | 0.113 | 0.112 | 0.108 | 0.207* | 0.107 |
|  | (0.071) | (0.071) | (0.071) | (0.071) | (0.071) | (0.071) | (0.070) | (0.072) | (0.071) | (0.072) | (0.085) | (0.071) |
| LA COVID-19 case rate | -0.000 | -0.000 | -0.000+ | -0.000 | -0.000 | -0.000 | -0.000 | -0.000 | -0.000 | -0.000 | -0.000 | -0.003 |
|  | (0.000) | (0.000) | (0.000) | (0.000) | (0.000) | (0.000) | (0.000) | (0.000) | (0.000) | (0.000) | (0.000) | (0.003) |
| baseline - Survey wave 2017-18 | ref. | ref. | ref. | ref. | ref. | ref. | ref. | ref. | ref. | ref. | ref. | ref. |
| August 2020 | 1.020 | 1.137 | 0.796 | 0.988 | 0.953 | 0.845 | 0.982 | 0.847 | 1.450 | 0.278 | 2.457 | 1.031 |
|  | (1.487) | (1.476) | (1.647) | (1.486) | (1.480) | (1.511) | (1.510) | (1.517) | (1.510) | (1.852) | (1.657) | (1.488) |
| November 2020 | 3.948* | 3.853* | 3.840* | 3.911* | 3.966* | 3.664* | 3.905* | 3.443* | 4.232** | 4.017* | 5.115** | 3.991* |
|  | (1.607) | (1.625) | (1.668) | (1.604) | (1.648) | (1.661) | (1.630) | (1.630) | (1.595) | (1.911) | (1.666) | (1.609) |
| March 2021 | 4.825** | 5.102** | 4.559** | 4.907** | 4.998** | 4.700** | 4.577** | 4.417** | 5.315** | 4.960* | 6.440*** | 4.780** |
|  | (1.646) | (1.654) | (1.763) | (1.644) | (1.654) | (1.780) | (1.660) | (1.683) | (1.642) | (1.926) | (1.791) | (1.650) |
| *Social capital measures* |  |  |  |  |  |  |  |  |  |  |  |  |
| Eating family meal (F) | -0.272 | -0.269 | -0.269 | -0.268 | -0.274 | -0.284 | -0.274 | -0.266 | -0.272 | -0.274 | -0.273 | -0.271 |
|  | (0.173) | (0.172) | (0.174) | (0.174) | (0.172) | (0.174) | (0.174) | (0.173) | (0.172) | (0.172) | (0.173) | (0.172) |
| August 2020 * Eating family meal (F) | -0.065 | -0.083 | -0.066 | -0.081 | -0.066 | -0.074 | -0.060 | -0.080 | -0.064 | -0.067 | -0.061 | -0.066 |
|  | (0.225) | (0.223) | (0.229) | (0.223) | (0.222) | (0.225) | (0.227) | (0.226) | (0.222) | (0.226) | (0.225) | (0.225) |
| November 2020 * Eating family meal (F) | -0.010 | -0.006 | -0.015 | -0.008 | -0.002 | 0.014 | -0.033 | -0.002 | -0.003 | -0.007 | -0.008 | -0.009 |
|  | (0.191) | (0.189) | (0.192) | (0.192) | (0.191) | (0.193) | (0.192) | (0.193) | (0.188) | (0.191) | (0.190) | (0.190) |
| March 2021 * Eating family meal (F) | -0.278 | -0.302 | -0.290 | -0.290 | -0.270 | -0.242 | -0.261 | -0.282 | -0.293 | -0.277 | -0.275 | -0.275 |
|  | (0.207) | (0.205) | (0.208) | (0.208) | (0.206) | (0.209) | (0.212) | (0.208) | (0.207) | (0.207) | (0.205) | (0.207) |
| Close friends (N) | 0.019 | 0.018 | 0.020 | 0.019 | 0.020 | 0.019 | 0.023 | 0.016 | 0.022 | 0.019 | 0.019 | 0.018 |
|  | (0.032) | (0.032) | (0.032) | (0.032) | (0.032) | (0.032) | (0.032) | (0.033) | (0.032) | (0.032) | (0.032) | (0.032) |
| August 2020 * Close friends (N) | -0.024 | -0.024 | -0.030 | -0.025 | -0.027 | -0.024 | -0.025 | -0.021 | -0.024 | -0.024 | -0.025 | -0.024 |
|  | (0.036) | (0.036) | (0.036) | (0.035) | (0.036) | (0.036) | (0.036) | (0.036) | (0.035) | (0.036) | (0.036) | (0.036) |
| November 2020 * Close friends (N) | 0.002 | 0.003 | 0.001 | 0.001 | 0.001 | 0.003 | -0.004 | 0.005 | -0.000 | 0.002 | 0.001 | 0.002 |
|  | (0.040) | (0.040) | (0.041) | (0.040) | (0.040) | (0.040) | (0.040) | (0.041) | (0.040) | (0.040) | (0.040) | (0.040) |
| March 2021 * Close friends (N) | -0.004 | -0.004 | -0.006 | -0.004 | -0.005 | -0.001 | -0.005 | -0.001 | -0.006 | -0.004 | -0.004 | -0.003 |
|  | (0.039) | (0.039) | (0.039) | (0.039) | (0.039) | (0.039) | (0.039) | (0.040) | (0.039) | (0.039) | (0.039) | (0.039) |
| Family support | -1.079** | -1.077** | -1.096** | -1.069** | -1.094** | -1.079** | -1.061** | -1.071** | -1.107** | -1.074** | -1.051** | -1.077** |
|  | (0.379) | (0.378) | (0.377) | (0.374) | (0.381) | (0.385) | (0.378) | (0.374) | (0.377) | (0.378) | (0.374) | (0.378) |
| August 2020 * Family support | 0.561 | 0.548 | 0.641 | 0.557 | 0.622 | 0.583 | 0.553 | 0.576 | 0.549 | 0.546 | 0.515 | 0.559 |
|  | (0.498) | (0.496) | (0.497) | (0.492) | (0.497) | (0.503) | (0.499) | (0.499) | (0.493) | (0.499) | (0.494) | (0.499) |
| November 2020 * Family support | -0.763 | -0.748 | -0.753 | -0.766 | -0.740 | -0.748 | -0.764 | -0.779 | -0.752 | -0.766 | -0.798+ | -0.768 |
|  | (0.481) | (0.482) | (0.480) | (0.474) | (0.484) | (0.484) | (0.484) | (0.474) | (0.475) | (0.481) | (0.473) | (0.482) |
| March 2021 * Family support | -0.585 | -0.642 | -0.617 | -0.631 | -0.598 | -0.605 | -0.629 | -0.585 | -0.615 | -0.587 | -0.632 | -0.578 |
|  | (0.520) | (0.520) | (0.511) | (0.514) | (0.519) | (0.533) | (0.521) | (0.517) | (0.516) | (0.518) | (0.516) | (0.520) |
| Friend support | -0.609* | -0.607* | -0.618* | -0.609* | -0.619* | -0.625* | -0.617* | -0.604* | -0.598* | -0.612* | -0.606* | -0.611* |
|  | (0.301) | (0.301) | (0.306) | (0.305) | (0.307) | (0.302) | (0.303) | (0.304) | (0.300) | (0.303) | (0.298) | (0.302) |
| August 2020 * Friend support | -0.184 | -0.189 | -0.147 | -0.197 | -0.191 | -0.147 | -0.177 | -0.200 | -0.157 | -0.182 | -0.193 | -0.183 |
|  | (0.394) | (0.394) | (0.398) | (0.399) | (0.399) | (0.397) | (0.395) | (0.399) | (0.384) | (0.397) | (0.391) | (0.394) |
| November 2020 * Friend support | -0.294 | -0.296 | -0.283 | -0.289 | -0.282 | -0.282 | -0.286 | -0.310 | -0.298 | -0.289 | -0.299 | -0.291 |
|  | (0.365) | (0.365) | (0.369) | (0.368) | (0.369) | (0.366) | (0.368) | (0.370) | (0.358) | (0.368) | (0.362) | (0.366) |
| March 2021 * Friend support | -0.170 | -0.172 | -0.177 | -0.164 | -0.154 | -0.186 | -0.165 | -0.169 | -0.164 | -0.165 | -0.182 | -0.163 |
|  | (0.391) | (0.391) | (0.383) | (0.392) | (0.393) | (0.390) | (0.392) | (0.394) | (0.383) | (0.393) | (0.388) | (0.392) |
| Neighbourhood safety | 0.121 | 0.118 | 0.124 | 0.123 | 0.122 | 0.117 | 0.107 | 0.027 | 0.137 | 0.119 | 0.120 | 0.122 |
|  | (0.162) | (0.162) | (0.160) | (0.161) | (0.161) | (0.161) | (0.163) | (0.174) | (0.162) | (0.162) | (0.163) | (0.161) |
| August 2020 * Neighbourhood safety | -0.521** | -0.517** | -0.533** | -0.530** | -0.523** | -0.524** | -0.515** | -0.481* | -0.522** | -0.519** | -0.524** | -0.523** |
|  | (0.185) | (0.184) | (0.183) | (0.185) | (0.185) | (0.186) | (0.187) | (0.201) | (0.186) | (0.185) | (0.186) | (0.185) |
| November 2020 * Neighbourhood safety | -0.333+ | -0.328+ | -0.339+ | -0.333+ | -0.335+ | -0.323+ | -0.315+ | -0.171 | -0.352+ | -0.331+ | -0.336+ | -0.330+ |
|  | (0.181) | (0.181) | (0.178) | (0.181) | (0.180) | (0.181) | (0.181) | (0.199) | (0.182) | (0.181) | (0.182) | (0.181) |
| March 2021 * Neighbourhood safety | -0.501** | -0.494** | -0.483** | -0.495** | -0.496** | -0.493** | -0.489* | -0.387+ | -0.473* | -0.498** | -0.505** | -0.499** |
|  | (0.191) | (0.190) | (0.187) | (0.190) | (0.189) | (0.187) | (0.191) | (0.212) | (0.191) | (0.191) | (0.193) | (0.191) |
| HH social capital | 0.060 | 0.067 | 0.057 | 0.068 | 0.057 | 0.057 | 0.053 | 0.055 | 0.053 | 0.058 | 0.071 | 0.061 |
|  | (0.113) | (0.115) | (0.115) | (0.110) | (0.113) | (0.114) | (0.115) | (0.113) | (0.114) | (0.115) | (0.113) | (0.113) |
| August 2020 * HH Social capital | -0.367+ | -0.388+ | -0.365+ | -0.389* | -0.362+ | -0.363+ | -0.369+ | -0.352+ | -0.359+ | -0.375+ | -0.367+ | -0.368+ |
|  | (0.194) | (0.198) | (0.192) | (0.192) | (0.193) | (0.194) | (0.196) | (0.194) | (0.194) | (0.194) | (0.193) | (0.194) |
| November 2020 * HH Social capital | -0.124 | -0.128 | -0.110 | -0.125 | -0.113 | -0.120 | -0.139 | -0.166 | -0.125 | -0.120 | -0.124 | -0.123 |
|  | (0.226) | (0.228) | (0.225) | (0.223) | (0.220) | (0.224) | (0.232) | (0.223) | (0.228) | (0.226) | (0.226) | (0.226) |
| March 2021 * HH Social capital | -0.211 | -0.232 | -0.199 | -0.230 | -0.205 | -0.210 | -0.188 | -0.229 | -0.190 | -0.206 | -0.206 | -0.217 |
|  | (0.215) | (0.220) | (0.214) | (0.213) | (0.213) | (0.212) | (0.216) | (0.215) | (0.217) | (0.216) | (0.215) | (0.215) |
| LA Relationships Index | -0.336 | -0.325 | -0.337 | -0.334 | -0.328 | -0.351 | -0.358 | -0.310 | -0.336 | -0.357 | -0.177 | -0.335 |
|  | (0.278) | (0.277) | (0.279) | (0.279) | (0.279) | (0.279) | (0.276) | (0.280) | (0.279) | (0.309) | (0.297) | (0.279) |
| August 2020 * LA Relationships Index | 0.345+ | 0.326+ | 0.366* | 0.326+ | 0.340+ | 0.340+ | 0.343+ | 0.338+ | 0.333+ | 0.580+ | 0.029 | 0.346+ |
|  | (0.185) | (0.183) | (0.183) | (0.185) | (0.186) | (0.184) | (0.189) | (0.186) | (0.185) | (0.344) | (0.256) | (0.186) |
| November 2020 * LA Relationships Index | 0.241+ | 0.249+ | 0.244+ | 0.244+ | 0.242+ | 0.245+ | 0.259+ | 0.204 | 0.230 | 0.213 | -0.013 | 0.239 |
|  | (0.145) | (0.147) | (0.145) | (0.146) | (0.145) | (0.144) | (0.149) | (0.150) | (0.145) | (0.259) | (0.236) | (0.145) |
| March 2021 * LA Relationships Index | 0.192 | 0.164 | 0.184 | 0.181 | 0.193 | 0.203 | 0.219 | 0.163 | 0.174 | 0.144 | -0.172 | 0.193 |
|  | (0.174) | (0.174) | (0.174) | (0.174) | (0.174) | (0.174) | (0.176) | (0.179) | (0.174) | (0.298) | (0.285) | (0.174) |
| Extra-curricular (N) | 0.176 | 0.184 | 0.174 | 0.178 | 0.171 | 0.169 | 0.173 | 0.162 | 0.175 | 0.178 | 0.196 | 0.176 |
|  | (0.161) | (0.160) | (0.162) | (0.161) | (0.161) | (0.161) | (0.160) | (0.161) | (0.161) | (0.160) | (0.162) | (0.161) |
| August 2020 * Extra-curricular (N) | -0.142 | -0.161 | -0.102 | -0.138 | -0.123 | -0.121 | -0.129 | -0.128 | -0.158 | -0.158 | -0.162 | -0.142 |
|  | (0.173) | (0.175) | (0.176) | (0.173) | (0.175) | (0.170) | (0.172) | (0.173) | (0.174) | (0.172) | (0.173) | (0.173) |
| November 2020 * Extra-curricular (N) | -0.329* | -0.328* | -0.326+ | -0.326+ | -0.317+ | -0.326* | -0.337* | -0.323* | -0.322+ | -0.329* | -0.346* | -0.328* |
|  | (0.165) | (0.167) | (0.168) | (0.166) | (0.168) | (0.166) | (0.164) | (0.164) | (0.165) | (0.164) | (0.164) | (0.165) |
| March 2021 *Extra-curricular (N) | -0.188 | -0.209 | -0.197 | -0.198 | -0.189 | -0.199 | -0.198 | -0.175 | -0.221 | -0.184 | -0.207 | -0.186 |
|  | (0.194) | (0.192) | (0.195) | (0.193) | (0.194) | (0.192) | (0.193) | (0.192) | (0.194) | (0.192) | (0.193) | (0.194) |
| August 2020 * Degree in HH |  | 0.301 |  |  |  |  |  |  |  |  |  |  |
|  |  | (0.349) |  |  |  |  |  |  |  |  |  |  |
| November 2020 * Degree in HH |  | -0.050 |  |  |  |  |  |  |  |  |  |  |
|  |  | (0.344) |  |  |  |  |  |  |  |  |  |  |
| March 2021 * Degree in HH |  | 0.374 |  |  |  |  |  |  |  |  |  |  |
|  |  | (0.346) |  |  |  |  |  |  |  |  |  |  |
| August 2020 * 1 employed in HH |  |  | 0.063 |  |  |  |  |  |  |  |  |  |
|  |  |  | (0.825) |  |  |  |  |  |  |  |  |  |
| August 2020 * 2+ employed in HH |  |  | -0.513 |  |  |  |  |  |  |  |  |  |
|  |  |  | (0.758) |  |  |  |  |  |  |  |  |  |
| November 2020 * 1 employed in HH |  |  | 0.060 |  |  |  |  |  |  |  |  |  |
|  |  |  | (0.767) |  |  |  |  |  |  |  |  |  |
| November 2020 * 2+ employed in HH |  |  | 0.092 |  |  |  |  |  |  |  |  |  |
|  |  |  | (0.724) |  |  |  |  |  |  |  |  |  |
| March 2021 * 1 employed in HH |  |  | 0.608 |  |  |  |  |  |  |  |  |  |
|  |  |  | (0.843) |  |  |  |  |  |  |  |  |  |
| March 2021 * 2+ employed in HH |  |  | 0.426 |  |  |  |  |  |  |  |  |  |
|  |  |  | (0.787) |  |  |  |  |  |  |  |  |  |
| August 2020 * HH coping well financially |  |  |  | 0.331 |  |  |  |  |  |  |  |  |
|  |  |  |  | (0.439) |  |  |  |  |  |  |  |  |
| November 2020 * HH coping well financially |  |  |  | 0.031 |  |  |  |  |  |  |  |  |
|  |  |  |  | (0.378) |  |  |  |  |  |  |  |  |
| March 2021 * HH coping well financially |  |  |  | 0.290 |  |  |  |  |  |  |  |  |
|  |  |  |  | (0.454) |  |  |  |  |  |  |  |  |
| August 2020 * Partner in HH |  |  |  |  | -0.159 |  |  |  |  |  |  |  |
|  |  |  |  |  | (0.534) |  |  |  |  |  |  |  |
| November 2020 * Partner in HH |  |  |  |  | -0.224 |  |  |  |  |  |  |  |
|  |  |  |  |  | (0.489) |  |  |  |  |  |  |  |
| March 2021 * Partner in HH |  |  |  |  | -0.212 |  |  |  |  |  |  |  |
|  |  |  |  |  | (0.487) |  |  |  |  |  |  |  |
| August 2020 * Health limits a little |  |  |  |  |  | 0.319 |  |  |  |  |  |  |
|  |  |  |  |  |  | (0.589) |  |  |  |  |  |  |
| August 2020 * Health limits a lot |  |  |  |  |  | -0.039 |  |  |  |  |  |  |
|  |  |  |  |  |  | (0.982) |  |  |  |  |  |  |
| November 2020 * Health limits a little |  |  |  |  |  | 0.217 |  |  |  |  |  |  |
|  |  |  |  |  |  | (0.508) |  |  |  |  |  |  |
| November 2020 * Health limits a lot |  |  |  |  |  | 1.338 |  |  |  |  |  |  |
|  |  |  |  |  |  | (1.159) |  |  |  |  |  |  |
| March 2021 * Health limits a little |  |  |  |  |  | -0.162 |  |  |  |  |  |  |
|  |  |  |  |  |  | (0.605) |  |  |  |  |  |  |
| March 2021 * Health limits a lot |  |  |  |  |  | 1.596 |  |  |  |  |  |  |
|  |  |  |  |  |  | (1.629) |  |  |  |  |  |  |
| August 2020 * Ethnic Mixed |  |  |  |  |  |  | 0.219 |  |  |  |  |  |
|  |  |  |  |  |  |  | (0.553) |  |  |  |  |  |
| August 2020 * Ethnic Asian |  |  |  |  |  |  | -0.219 |  |  |  |  |  |
|  |  |  |  |  |  |  | (0.536) |  |  |  |  |  |
| August 2020 * Ethnic Other |  |  |  |  |  |  | 0.363 |  |  |  |  |  |
|  |  |  |  |  |  |  | (1.307) |  |  |  |  |  |
| August 2020 * Ethnic Black |  |  |  |  |  |  | 0.177 |  |  |  |  |  |
|  |  |  |  |  |  |  | (1.106) |  |  |  |  |  |
| November 2020 * Mixed |  |  |  |  |  |  | 1.328* |  |  |  |  |  |
|  |  |  |  |  |  |  | (0.566) |  |  |  |  |  |
| November 2020 * Ethnic Asian |  |  |  |  |  |  | 0.021 |  |  |  |  |  |
|  |  |  |  |  |  |  | (0.417) |  |  |  |  |  |
| November 2020 * Ethnic Other |  |  |  |  |  |  | 1.139 |  |  |  |  |  |
|  |  |  |  |  |  |  | (1.432) |  |  |  |  |  |
| November 2020 * Ethnic Black |  |  |  |  |  |  | -0.521 |  |  |  |  |  |
|  |  |  |  |  |  |  | (0.829) |  |  |  |  |  |
| March 2021 * Ethnic Mixed |  |  |  |  |  |  | 0.839 |  |  |  |  |  |
|  |  |  |  |  |  |  | (0.599) |  |  |  |  |  |
| March 2021 * Ethnic Asian |  |  |  |  |  |  | 0.374 |  |  |  |  |  |
|  |  |  |  |  |  |  | (0.578) |  |  |  |  |  |
| March 2021 * Ethnic Other |  |  |  |  |  |  | 1.390 |  |  |  |  |  |
|  |  |  |  |  |  |  | (1.272) |  |  |  |  |  |
| March 2021 * Ethnic Black |  |  |  |  |  |  | 0.991 |  |  |  |  |  |
|  |  |  |  |  |  |  | (0.854) |  |  |  |  |  |
| August 2020 * Female |  |  |  |  |  |  |  | 0.115 |  |  |  |  |
|  |  |  |  |  |  |  |  | (0.328) |  |  |  |  |
| November 2020 * Female |  |  |  |  |  |  |  | 0.785* |  |  |  |  |
|  |  |  |  |  |  |  |  | (0.336) |  |  |  |  |
| March 2021 * Female |  |  |  |  |  |  |  | 0.496 |  |  |  |  |
|  |  |  |  |  |  |  |  | (0.368) |  |  |  |  |
| August 2020 * Age 12-13 |  |  |  |  |  |  |  |  | -0.840+ |  |  |  |
|  |  |  |  |  |  |  |  |  | (0.440) |  |  |  |
| August 2020 * Age 14-15 |  |  |  |  |  |  |  |  | -1.109 |  |  |  |
|  |  |  |  |  |  |  |  |  | (0.789) |  |  |  |
| November 2020 * Age 12-13 |  |  |  |  |  |  |  |  | -0.409 |  |  |  |
|  |  |  |  |  |  |  |  |  | (0.408) |  |  |  |
| November 2020 * Age 14-15 |  |  |  |  |  |  |  |  | -0.862 |  |  |  |
|  |  |  |  |  |  |  |  |  | (0.767) |  |  |  |
| March 2021 * Age 12-13 |  |  |  |  |  |  |  |  | -0.575 |  |  |  |
|  |  |  |  |  |  |  |  |  | (0.454) |  |  |  |
| March 2021 * Age 14-15 |  |  |  |  |  |  |  |  | -1.305+ |  |  |  |
|  |  |  |  |  |  |  |  |  | (0.767) |  |  |  |
| August 2020 * LA Economic disadvantage |  |  |  |  |  |  |  |  |  | 0.283 |  |  |
|  |  |  |  |  |  |  |  |  |  | (0.325) |  |  |
| November 2020 * LA Economic disadvantage |  |  |  |  |  |  |  |  |  | -0.031 |  |  |
|  |  |  |  |  |  |  |  |  |  | (0.259) |  |  |
| March 2021 * LA Economic disadvantage |  |  |  |  |  |  |  |  |  | -0.054 |  |  |
|  |  |  |  |  |  |  |  |  |  | (0.296) |  |  |
| August 2020 * LA Social disadvantage |  |  |  |  |  |  |  |  |  |  | -0.479+ |  |
|  |  |  |  |  |  |  |  |  |  |  | (0.278) |  |
| November 2020 * LA Social disadvantage |  |  |  |  |  |  |  |  |  |  | -0.387 |  |
|  |  |  |  |  |  |  |  |  |  |  | (0.282) |  |
| March 2021 * LA Social disadvantage |  |  |  |  |  |  |  |  |  |  | -0.563+ |  |
|  |  |  |  |  |  |  |  |  |  |  | (0.309) |  |
| August 2020 * LA Social disadvantage-sq. |  |  |  |  |  |  |  |  |  |  | -0.166+ |  |
|  |  |  |  |  |  |  |  |  |  |  | (0.093) |  |
| November 2020 * LA Social disadvantage-sq. |  |  |  |  |  |  |  |  |  |  | -0.144+ |  |
|  |  |  |  |  |  |  |  |  |  |  | (0.088) |  |
| March 2021 * LA Social disadvantage-sq. |  |  |  |  |  |  |  |  |  |  | -0.183* |  |
|  |  |  |  |  |  |  |  |  |  |  | (0.090) |  |
| August 2020 * LA COVID-19 case rate |  |  |  |  |  |  |  |  |  |  |  | 0.003 |
|  |  |  |  |  |  |  |  |  |  |  |  | (0.003) |
| November 2020 * LA COVID-19 case rate |  |  |  |  |  |  |  |  |  |  |  | 0.004 |
|  |  |  |  |  |  |  |  |  |  |  |  | (0.005) |
| March 2021 * LA COVID-19 case rate |  |  |  |  |  |  |  |  |  |  |  | 0.000 |
|  |  |  |  |  |  |  |  |  |  |  |  |  |
| Constant | 11.376*** | 11.352*** | 11.499*** | 11.394*** | 11.320*** | 11.532*** | 11.533*** | 11.744*** | 11.223*** | 11.455*** | 10.561*** | 11.367*** |
|  | (1.616) | (1.617) | (1.701) | (1.622) | (1.640) | (1.630) | (1.619) | (1.626) | (1.615) | (1.744) | (1.617) | (1.617) |
| Observations | 3176 | 3176 | 3176 | 3176 | 3176 | 3176 | 3176 | 3176 | 3176 | 3176 | 3176 | 3176 |

*Notes*: F=Frequency; N=Number; HH=Household; LA=Local Authority; SDQ=Strengths and Difficulties Questionnaire; Ext.=Externalizing scores; Sq.=squared; UK Household Longitudinal Study Mainstage and UK Household Longitudinal Study COVID-19 data; standard errors in parentheses.

+ p<.10, * p<.05, ** p<.01, *** p<.001

**Table S.8 – Testing whether internalising and externalising scores moderate the association between survey-periods and social capital; fixed effects analysis**

|  | Model 1 | Model 2 | Model 3 | Model 4 | Model 5 | Model 6 | Model 7 | Model 8 | Model 9 | Model 10 |
| --- | --- | --- | --- | --- | --- | --- | --- | --- | --- | --- |
| Outcome | SDQ Int. | Family Meals | Close friends | Family support | Neigh. safety | SDQ Ext. | Family Meals | Close friends | Family support | Neigh. safety |
|  |  |  |  |  |  |  |  |  |  |  |
| baseline - Survey wave 2017-18 | ref. | ref. | ref. | ref. | ref. | ref. | ref. | ref. | ref. | ref. |
| November 2020 | 7.930*** | 0.109 | -0.915 | -0.021 | -0.118 | 4.259** | -0.154 | -0.859 | -0.021 | -0.370 |
|  | (1.874) | (0.129) | (1.501) | (0.078) | (0.232) | (1.378) | (0.104) | (1.295) | (0.055) | (0.296) |
| Eating family meal (F) | -0.726* |  |  |  |  | -0.165 |  |  |  |  |
|  | (0.325) |  |  |  |  | (0.214) |  |  |  |  |
| November 2020 * Family meal (F) | -0.342 |  |  |  |  | -0.023 |  |  |  |  |
|  | (0.325) |  |  |  |  | (0.242) |  |  |  |  |
| Close friends (N) | -0.030 |  |  |  |  | 0.014 |  |  |  |  |
|  | (0.021) |  |  |  |  | (0.013) |  |  |  |  |
| November 2020 * Close friends (N) | -0.122* |  |  |  |  | -0.042 |  |  |  |  |
|  | (0.051) |  |  |  |  | (0.034) |  |  |  |  |
| Family support | 0.879 |  |  |  |  | 0.081 |  |  |  |  |
|  | (0.752) |  |  |  |  | (0.412) |  |  |  |  |
| November 2020 * Family support | -1.474* |  |  |  |  | -0.412 |  |  |  |  |
|  | (0.616) |  |  |  |  | (0.441) |  |  |  |  |
| Neighbourhood safety | -0.156 |  |  |  |  | 0.655 |  |  |  |  |
|  | (0.231) |  |  |  |  | (0.217) |  |  |  |  |
| November 2020 * Neigh. safety | -0.211 |  |  |  |  | -0.758* |  |  |  |  |
|  | (0.267) |  |  |  |  | (0.295) |  |  |  |  |
| SDQ Internalising |  | -0.055* | -0.360+ | 0.010 | -0.038 |  |  |  |  |  |
|  |  | (0.023) | (0.190) | (0.020) | (0.025) |  |  |  |  |  |
| November 2020 * SDQ Internal |  | -0.022 | -0.102 | -0.013 | 0.040 |  |  |  |  |  |
|  |  | (0.021) | (0.216) | (0.011) | (0.025) |  |  |  |  |  |
| SDQ Externalising |  |  |  |  |  |  | -0.034 | 0.225 | 0.004 | -0.021 |
|  |  |  |  |  |  |  | (0.022) | (0.212) | (0.015) | (0.022) |
| November 2020 * SDQ External |  |  |  |  |  |  | 0.015 | -0.226 | -0.011 | 0.076* |
|  |  |  |  |  |  |  |  |  |  |  |
| Constant | 5.195* | 3.542*** | 10.558* | 2.765*** | 2.696*** | 3.772** | 3.498*** | 7.690+ | 2.781*** | 2.678*** |
|  | (2.268) | (0.346) | (4.723) | (0.227) | (0.260) | (1.440) | (0.377) | (4.501) | (0.200) | (0.257) |
| Observations | 1214 | 1214 | 1214 | 1214 | 1214 | 1214 | 1214 | 1214 | 1214 | 1214 |

*Notes*: F=Frequency; N=Number; HH=Household; LA=Local Authority; SDQ=Strengths and Difficulties Questionnaire; Int.=Internalizing scores; Ext.=Externalizing scores; Sq.=squared; UK Household Longitudinal Study Mainstage and UK Household Longitudinal Study COVID-19 data; standard errors in parentheses.

+ p<.10, * p<.05, ** p<.01, *** p<.001
